# Glutamine promotes escape from therapy-induced senescence in tumor cells

**DOI:** 10.18632/aging.203495

**Published:** 2021-09-07

**Authors:** Francesco Pacifico, Nadia Badolati, Stefano Mellone, Mariano Stornaiuolo, Antonio Leonardi, Elvira Crescenzi

**Affiliations:** 1 Istituto di Endocrinologia ed Oncologia Sperimentale, CNR, Naples 80131, Italy; 2 Department of Pharmacy, University of Naples Federico II, Naples 80149, Italy; 3 Dipartimento di Medicina Molecolare e Biotecnologie Mediche, University of Naples Federico II, Naples 80131, Italy

**Keywords:** therapy-induced senescence, escape, glutamine, glutamine synthetase, cancer stem cells

## Abstract

Therapy-induced senescence (TIS) is a major cellular response to anticancer therapies. While induction of a persistent growth arrest would be a desirable outcome in cancer therapy, it has been shown that, unlike normal cells, cancer cells are able to evade the senescence cell cycle arrest and to resume proliferation, likely contributing to tumor relapse. Notably, cells that escape from TIS acquire a plastic, stem cell-like phenotype. The metabolic dependencies of cells that evade senescence have not been thoroughly studied. In this study, we show that glutamine depletion inhibits escape from TIS in all cell lines studied, and reduces the stem cell subpopulation. In line with a metabolic reliance on glutamine, escaped clones overexpress the glutamine transporter SLC1A5. We also demonstrate a central role of glutamine synthetase that mediates resistance to glutamine deprivation, conferring independence from exogenous glutamine. Finally, rescue experiments demonstrate that glutamine provides nitrogen for nucleotides biosynthesis in cells that escape from TIS, but also suggest a critical involvement of glutamine in other metabolic and non-metabolic pathways. On the whole, these results reveal a metabolic vulnerability of cancer stem cells that recover proliferation after exposure to anticancer therapies, which could be exploited to prevent tumor recurrence.

## INTRODUCTION

Therapy-induced senescence (TIS) is a state of permanent cell cycle arrest which ensues in both normal and neoplastic cells upon cytotoxic chemotherapies or radiotherapies [1, 2]. Induction of TIS has been detected in cells in culture and, more importantly, in human tissues [3, 4]. Drug-induced senescent cells acquire distinctive phenotypic alterations, such as large and flat morphology, extensive vacuolization, increased expression and activity of senescence-associated beta-galactosidase (SA-β-gal), and the expression of specific cyclin-dependent kinase inhibitors, especially p21CIP1. In addition, TIS cells show an increased expression and secretion of pro-inflammatory cytokines, chemokines, matrix remodelling enzymes and growth factors, referred to as senescence-associated secretory phenotype (SASP) [5].

Unlike normal cells, it has been demonstrated that cancer cells are able to escape from TIS [6, 7], suggesting that senescence in tumor cells represents a state of prolonged but not permanent cell cycle arrest, after which cells may recover proliferation. Indeed, it has been proposed that TIS could reflect one form of tumor dormancy: cells that undergo a senescence growth arrest after treatments could persist in tissue over long periods of time and ultimately contribute to recurrence [8]. As such, escape from TIS might represent a challenge in cancer treatments, favoring relapse. In line with these observations, induction of senescence was shown to be associated with adverse treatment outcome and decreased overall survival in cancer patients [9–12].

Evasion from drug-induced senescence is a rare event and only a small subset of cancer cells is able to re-enter the cell cycle. Cells that evade TIS display a plastic, stem cell-like phenotype [13–15] characterized by the ability to self-renew and to give rise to a more differentiated progeny. Interestingly, a direct link between the induction of premature senescence and the reprogramming of cells into stem-like, tumor-initiating cells has been recently demonstrated, defined as senescence-associated stemness (SAS). Thus, enforcement of senescence-like growth arrest reprograms bulk tumor cells into cancer stem cells (CSC) [16]. CSC represent a functionally heterogeneous cell population. Different subsets of CSC have been identified within tumors that differ in cell cycle status, differentiation potential and expression of stem-specific markers. Notably, CSC have distinctive metabolic features as compared with their differentiated progenies and specific metabolic traits are required for maintenance of different subsets of CSC [17, 18].

Little is known about the mechanisms governing evasion from TIS in tumor cells and the metabolic needs of cells that escape from senescence. In this study we investigated the metabolic requirements of cells that escape from chemotherapy-induced senescence. We show that although parental cell lines analyzed show different metabolic characteristics, all cells rely on glutamine for escaping from TIS. Accordingly, modulating glutamine concentration in culture media results in increased or decreased evasion from senescence. CSC that evade senescence growth arrest overexpress the glutamine transporter SLC1A5. We also demonstrate that induction of glutamine synthetase (GS; glutamate-ammonia ligase) mediates resistance to glutamine ablation and allows evasion from TIS in glutamine-deprived conditions. Finally, our data support a role for glutamine in nucleotides biosynthesis in cells that escape from TIS, but also suggest a critical involvement of glutamine in other metabolic and non-metabolic pathways.

## RESULTS

**Figure 1:**
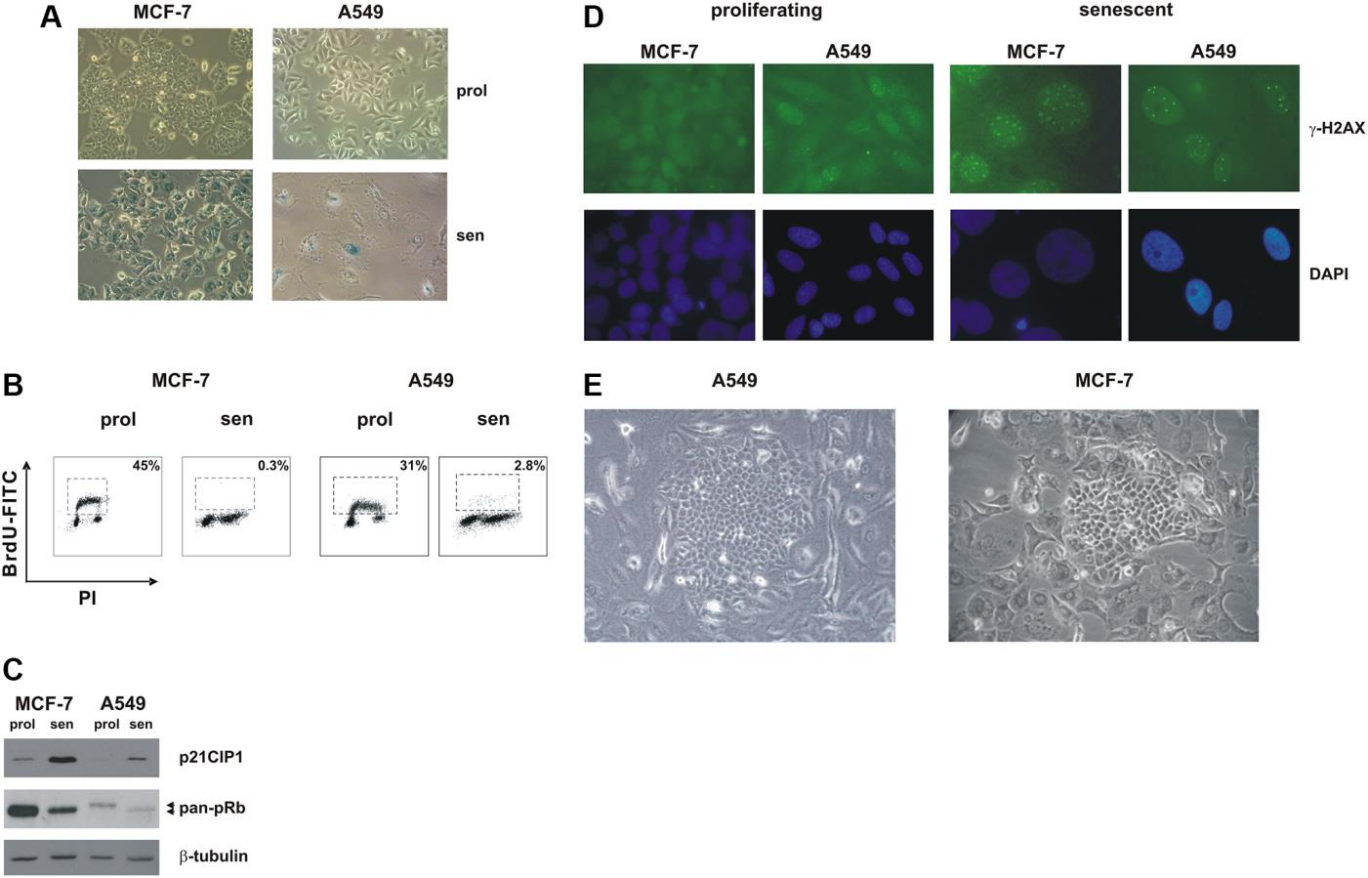
**Premature senescence in MCF-7 and A549 cells.** MCF-7 and A549 cells were treated with doxorubicin for 72 h. Cells were extensively washed and analyzed 7 days after release from the drug. (**A**) Morphological alterations and SA-β-gal staining in drug-induced senescent cells. Proliferating cells and doxorubicin-induced senescent cells were stained to detect SA-β-gal activity. Phase contrast microscopy images were captured using Canon powershot G6 camera at 10x magnification, 6× digital zoom. (**B**) Representative flow cytometric data. Proliferating and senescent MCF-7 and A549 cells were incubated with 5-bromo-2-deoxyuridine (BrdU), for 30 min and 1 hour, respectively. The number of BrdU-labelled cells was determined and the percentage is shown in the chart. (**C**) Accumulation of p21^CIP1^ and hypophosphorylated pRb protein in drug-induced senescent cells. Filters were stripped and reprobed with β-tubulin antibodies as a loading control. (**D**) Proliferating and doxorubicin-induced senescent MCF-7 and A549 cells were immunostained with an anti-γ-H2AX monoclonal antibody followed by secondary fluorescein-conjugate antibodies. Nuclei were stained with DAPI. Samples were visualized on a Zeiss Axioplan fluorescent microscope at 63x magnification. (**E**) Representative phase contrast microscopy images of clones that evade senescence; note the flat and enlarged morphology of TIS cells surrounding the escaped clone. Phase contrast microscopy images were captured using Canon powershot G6 camera at 20× magnification, 6× digital zoom.

In order to investigate metabolic requirements of cells that evade chemotherapy-induced senescence, we treated with sublethal concentrations of doxorubicin two wild-type p53-positive cancer cell line, MCF-7 (breast adenocarcinoma) and A549 (lung adenocarcinoma). These cells have been previously characterized as a model of TIS in our laboratory [19–21], and both are able to re-enter cell cycle from a senescent state [7, 21, 22]. As shown in Figure 1, MCF-7 and A549 cells readily underwent senescence upon treatment with doxorubicin and acquired a flat and enlarged morphology and positivity to SA-beta-gal staining (Figure 1A). Both cell lines were cell cycle arrested (Figure 1B), displayed upregulation of the cdk-inhibitor p21CIP1 and accumulation of hypophosphorylated pRb (Figure 1C). Furthermore, TIS cells accumulated persistent DNA damage foci, as shown by staining with γ-H2AX antibodies (Figure 1D).

In line with previous observations [7, 22] when TIS cells were kept in culture for prolonged periods of time (from 15 to 60 days) colonies that evaded the senescent growth arrest appeared (Figure 1E). A549 cells displayed a higher frequency of escape as compared to MCF-7 cells (1 in 1,650 ± 124 and ~1 in 15,000 ± 3500 cells, respectively, based on an initial seeding of 80,000 senescent cells in A549 and 120,000 senescent cells in MCF-7), and we also noticed that A549 escaped colonies arose after a shorter period of time, as compared to MCF-7 cells (18.5 d ± 4.1 and 25.8 d ± 7.3, respectively, *p* = 0.002, based on an initial seeding of 80,000 senescent cells in A549 and 120,000 senescent cells in MCF-7).

In order to confirm that senescence escape cells are characterized by a stem cell-like phenotype, we isolated clonal populations that emerge from senescent MCF-7 cells (hereafter defined as escaped clones) and analyzed the expression of CD44 and CD24 breast cancer stem cell markers [23] by flow cytometry. As reported in Table 1, all escaped clones analyzed showed an increased CD44^+^/CD24^−/low^ subpopulation, as compared to parental MCF-7 cells. The enrichment in CD44^+^/CD24^−/low^ cells varied among different clones, likely reflecting the relative proportion of CSC. As expected, escaped clones in culture progressively lost their stem-cell properties, spontaneously acquiring a more differentiated phenotype resembling the parental cell line. Hence, all the experiments were performed in clones within five passages in culture.

**Table 1:** CD44^+^/CD24^−/lo^^w^ subpopulation was estimated in parental MCF-7 cell line and in escaped clones.

	**CD44^+^/CD24** ^−**/low**^ **(%)**	**CD44^+^/CD24** ^−**/low**^ **(fold of MCF-7)**
MCF-7	3.5 ± 0.4	1
ESC 1	5.5 ± 1.2	1.57
ESC 2	5.1 ± 0.4	1.46
ESC 3	12.3 ± 2.2	3.51
ESC 4	11.8 ± 0.5	3.37
ESC 9	10 ± 0.85	2.86
ESC 10	29.4 ± 1.4	8.40

Column 1: Percentage of CD44^+^/CD24^−/low^ cells was analyzed by flow cytometry. Data are mean ± S.D. of three independent experiments. Column 2: Relative increase in CD44^+^/CD24^−/low^ cells in escaped clones as compared to MCF-7 cells.

**Figure 2:**
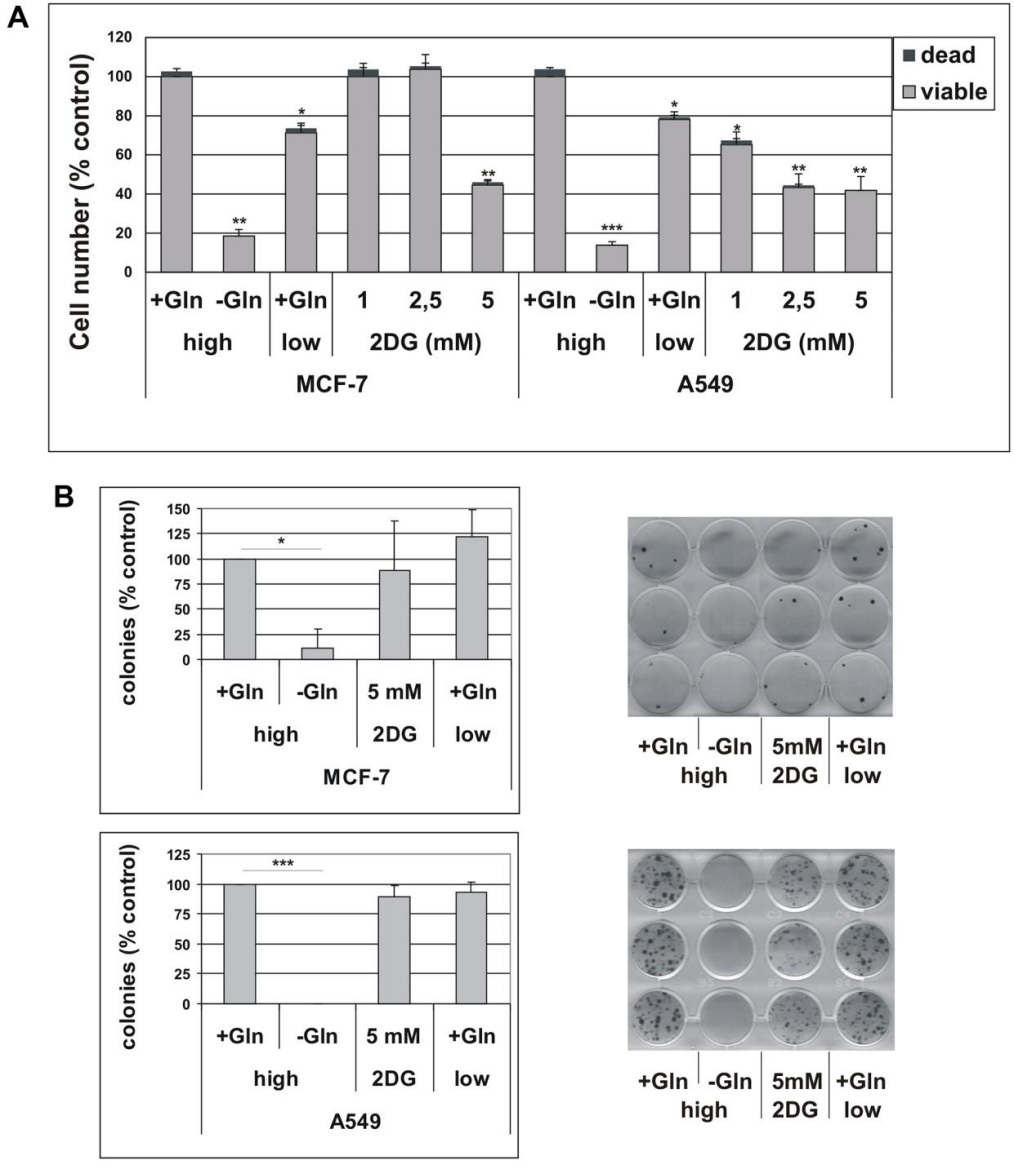
(**A**) Effect of glutamine deprivation on cell viability. MCF-7 and A549 cells were grown for 72 hours in either high-glucose (high) or low-glucose (low) medium, in the presence (+Gln) or in the absence (−Gln) of 2 mM glutamine or in the presence of indicated concentrations of 2-deoxyglucose (2DG). Cells were stained with trypan blue and counted. The percentage of dead, trypan blue-positive cells is shown. Data are mean ± S.D. of three independent experiments. (**B**) The effect of glutamine deprivation on TIS escape. Doxorubicin-induced senescent MCF-7 and A549 cells were grown in high-glucose (high) or low-glucose (low) medium, in the presence (+Gln) or in the absence (−Gln) of 2 mM glutamine or in the presence of 2-deoxyglucose (2DG). Colonies that evaded the senescent growth arrest were stained and counted. Left panels: quantification of the colony escape assay. Data are mean ± S.D. of three independent experiments. Right panels: representative images of colony escape assay.

One of the hallmarks of cancer cells is metabolic plasticity, meaning that distinct metabolic pathways can be activated in different subsets of cancer cells. Glucose and glutamine (Gln) are the two essential molecules metabolized to support bioenergetics and biosynthetic processes [24, 25]. Hence, we first characterized the response of parental cell lines to glucose or glutamine deprivation. As shown in Figure 2A, the number of A549 and MCF-7 cells was reduced by about 20% by low glucose concentration (1 g/L) in the medium, as compared to high glucose-containing medium (4.5 g/L). The addition of 2-deoxy-glucose (2DG), a glucose analog that specifically inhibits glycolysis, to culture medium also reduced cell proliferation over 72 hours, although a cell line-specific sensitivity was observed, with A549 cells being more sensitive than MCF-7 cells (Figure 2A). Glutamine-deprivation significantly reduced cell proliferation in both cell lines. The percentage of dead cells never exceeded 4% (Figure 2A). We confirmed that 72 h Gln starvation produced a marked decrease in the intracellular content of glutamine in both cell lines, as determined by liquid chromatography-mass spectrometry (LC−MS) analysis. After 72h of glutamine deprivation, the intracellular glutamine content in A549 cells decreased from 4.43 ± 0.28 to 1.21 ± 0.42 nmol/mg protein (*p* = 0.0004), and from 1.18 ± 0.17 to 0.43 ± 0.31 nmol/mg protein in MCF-7 cells (*p* = 0.0213).

**Figure 3:**
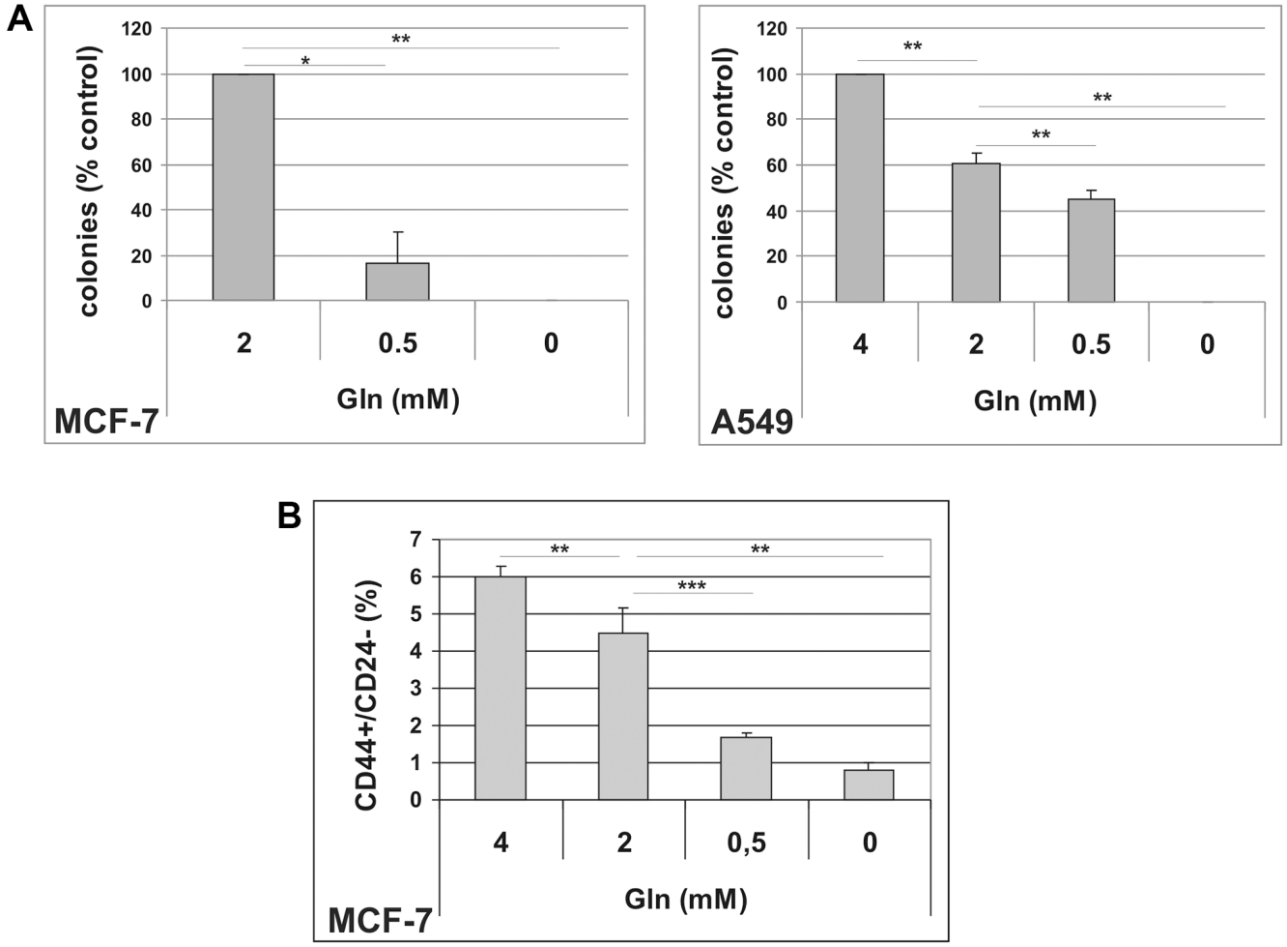
(**A**) Dose-dependent effects of glutamine on TIS escape. Doxorubicin-induced senescent MCF-7 and A549 cells were grown in media with different glutamine concentrations. Colonies that evaded the senescent growth arrest were stained and counted. Data are mean ± S.D. of three independent experiments. (**B**) Dose-dependent effects of glutamine on CD44^+^/CD24^−^ subpopulation. MCF-7 cells were grown for 48 hours in media with different glutamine concentrations. Expression of CD44 and CD24 was analyzed by flow cytometry. Data are mean ± S.D. of three independent experiments.

To get insight into the antiproliferative effects of glutamine deprivation, we analyzed the cell cycle distribution in A549 and MCF-7 cells grown in Gln-supplemented or Gln-deprived conditions for 72 h. Glutamine deprivation resulted in a significant increase in G2/M compartment in A549 cells, while in MCF-7 cells an increase in S-phase fraction was detected (Supplementary Figure 1A). These results were confirmed by analyses of BrdU incorporation, which showed a marked decrease in DNA synthesis, together with a significant increase in G2/M population in A549 cells, and a S-phase accumulation in MCF-7 cells (Supplementary Figure 1B). Notably, no increase in sub-G1, apoptotic cells, was observed in Gln-deprived conditions indicating that Gln-deprivation inhibits cell proliferation, but does not affect cell survival (Supplementary Figure 1C). Evidence for an important role of glutamine in the maintenance of cancer stem cells have been provided in several tumor types [26, 27]. Since both A549 and MCF-7 cells displayed higher sensitivity to Gln-deprivation than glucose-deprivation, we decided to investigate a potential role of glutamine metabolism in escape from TIS. In order to analyze metabolic needs of cells that evade senescence, we induced TIS in our cell lines, released them in complete medium to allow for cell attachment, and thereafter maintained them in either complete medium (2 mM Gln) or in Gln-deprived medium. In addition, effects of low glucose concentration and 2DG were investigated. As shown in Figure 2B, glutamine withdrawal reduced evasion from TIS in both MCF-7 and A549 cell lines, while long term exposure to low glucose or 5 mM 2DG did not affect the emergence of escaped colonies, thereby indicating that glutamine availability affects escape from senescence. Similar results were obtained after inducing TIS by treatment with the chemotherapeutic cisplatin in MCF-7 cells (Supplementary Figure 2). It is important to note that glutamine withdrawal did not alter development of TIS and that Gln-deprived senescent cells retained the senescent phenotype and the expression of senescence markers (Supplementary Figure 3). To further analyze the glutamine dependency of escaped cells, we maintained TIS cells in media with glutamine or with stepwise decrease in glutamine concentrations. In these conditions, we observed that the ability of cancer cells to escape from TIS decreased in parallel with the reduction in glutamine concentration (Figure 3A). Furthermore, increasing glutamine concentration to 4 mM increased the number of colonies that evade TIS (Figure 3A, A549). Interestingly, further increase in glutamine concentration, above 4 mM, led to no further enhancement of TIS escape. On the whole, these data demonstrate that glutamine depletion inhibits escape of CSC from TIS.

CSC are functionally heterogeneous and different subsets of CSC show different metabolic requirements [28]. Since mammary escaped clones showed an increased CD44^+^/CD24^−/low^ subpopulation (Table 1), we exposed parental MCF-7 cells to Gln-deprived medium, and analyzed the CD44^+^/CD24^−/low^ subpopulation by flow cytometry. As illustrated in Figure 3B and Supplementary Figure 4A, gradual reduction in glutamine concentration in culture media was accompanied by a parallel decrease in the percentage of the CD44^+^/CD24^−/low^ cells. In addition, increasing glutamine concentration to 4 mM resulted in a significant increase in CD44^+^/CD24^−/low^ cells. These data suggest that the CSC subset defined by CD44^+^/CD24^−/low^ expression in MCF-7 cells is largely glutamine-dependent and is involved in TIS evasion.

**Figure 4:**
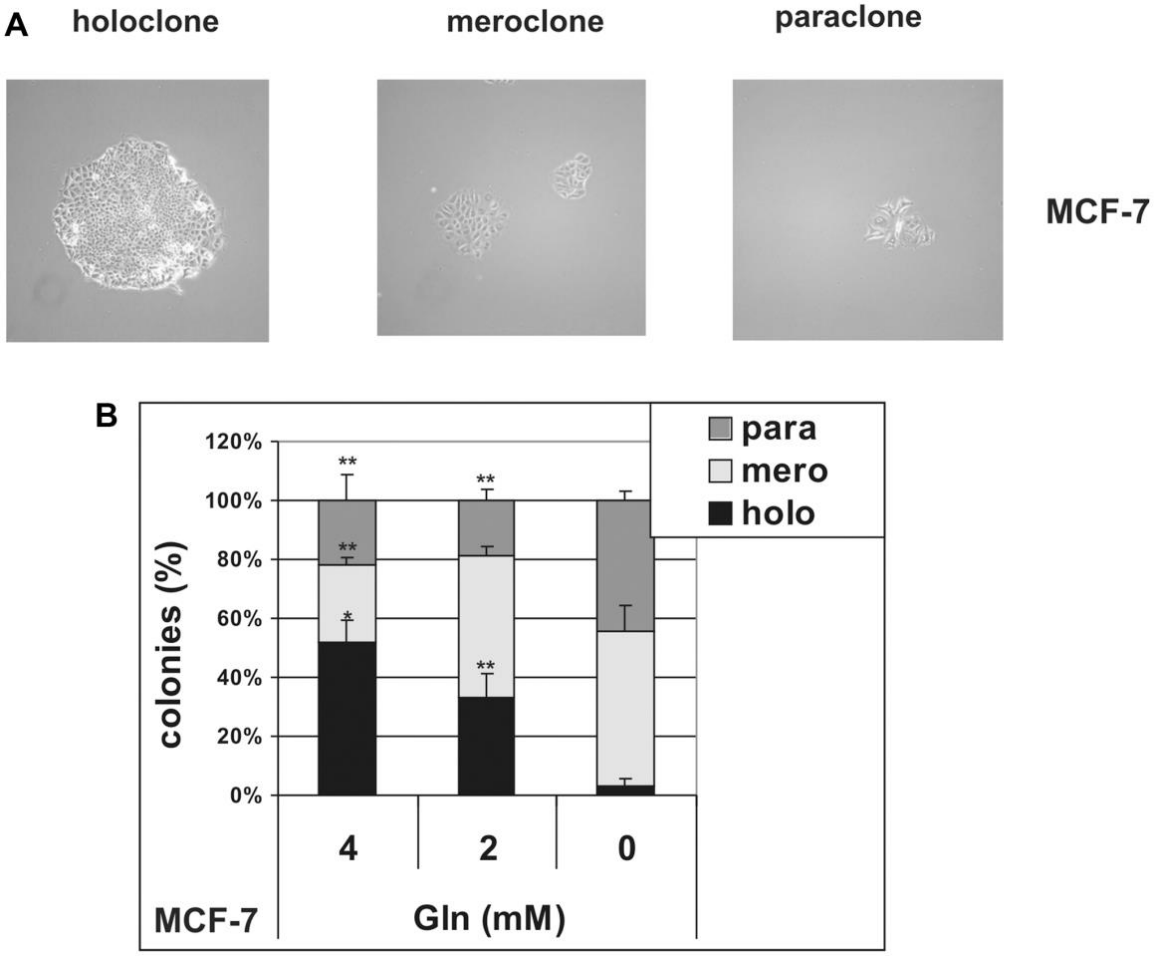
**Glutamine controls clonal heterogeneity in MCF-7 cells.** MCF-7 cells were cultured at low density in the presence (4 mM and 2 mM) or in the absence of glutamine. (**A**) Representative phase contrast microscopy images of holoclones, meroclones and paraclones. Holoclones are large colonies with compact and smooth borders, while paraclone are small colonies with flattened shape. An intermediate phenotype characterizes meroclones. Phase contrast microscopy images were captured using Canon powershot G6 camera at 10× magnification, 6× digital zoom. (**B**) After 10 days of culture, the number of holoclones, meroclones and paraclones were counted. Relative percentage of colonies is shown. Data are mean ± S.D. of three independent experiments.

To further investigate the effects of glutamine deprivation on stem cells, we analyzed the formation of holoclones, meroclones and paraclones in MCF-7 cells, plated as single cells in the presence or in the absence of glutamine. It has been demonstrated that when normal [29] and cancer [30, 31] cells are plated at low density, colonies derived from stem cells with the highest proliferative ability give rise to holoclones, while colonies derived from their differentiated progeny grow as small paraclones. Meroclones have an intermediate phenotype and are believed to be formed by transient-amplifying cells. Hence, these three types of colonies reflect the vertical hierarchy of CSC and are easily distinguished, being different in size and morphology (Figure 4A). As shown in Figure 4B, glutamine deprivation specifically suppressed the growth of holoclones, whereas increasing glutamine concentration to 4 mM led to a parallel increase in the relative percentage of holoclones, without changing average holoclone sizes (Supplementary Figure 5). These data demonstrate that stem cells, transient-amplifying cells and differentiated cells present in MCF-7 cell line show different metabolic requirements, and that stem cells rely on glutamine for holoclones formation.

**Figure 5:**
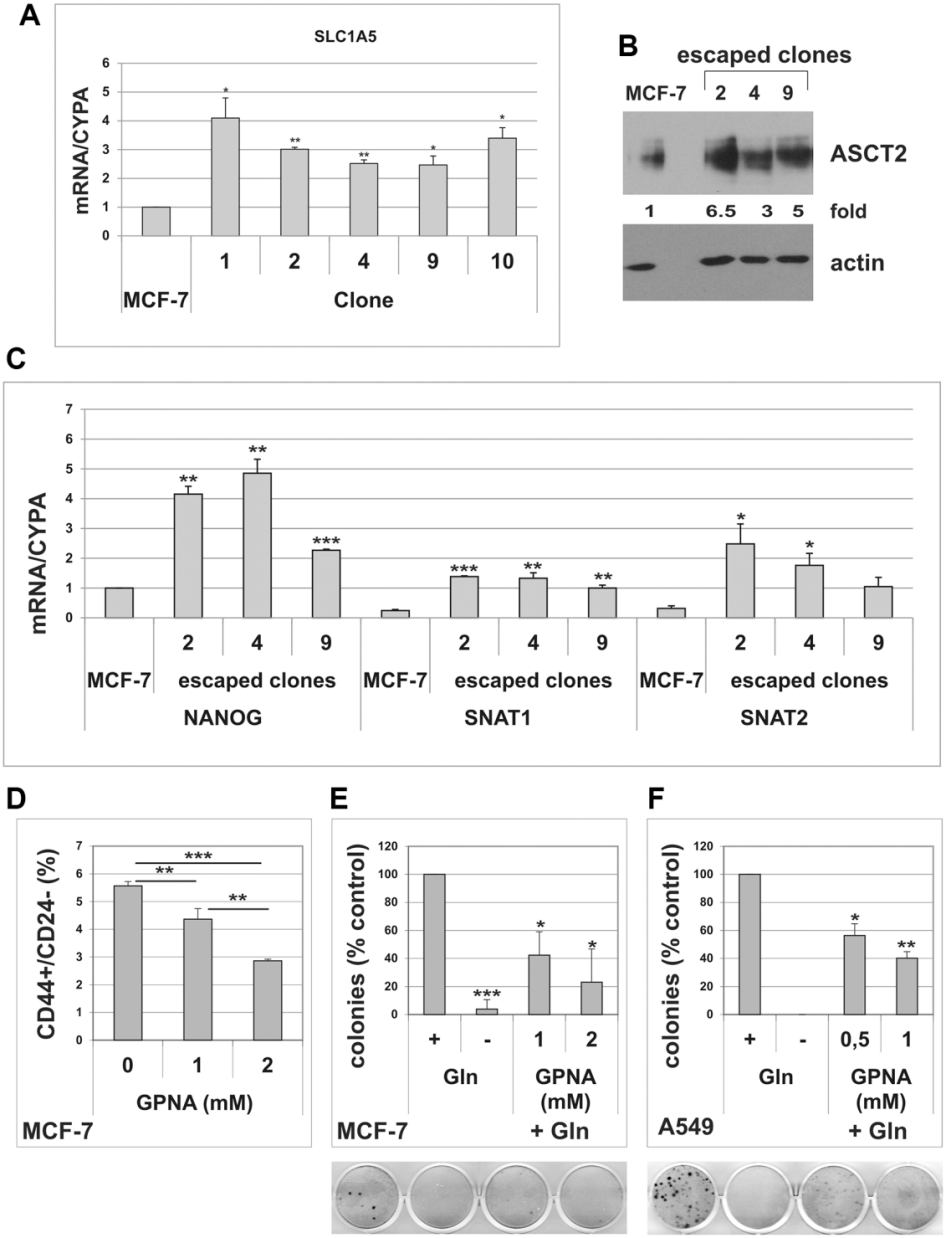
**Cells that escape from TIS overexpress the glutamine transporter SLC1A5.** (**A**) Escaped clones were isolated from senescent MCF-7 cells. Expression of SLC1A5 was analyzed by real-time PCR. Data are mean ± S.D. of three independent experiments. (**B**) Expression of SLC1A5 protein was analyzed in parental MCF-7 cells and in three escaped clones. Filters were stripped and reprobed with anti-actin antibodies as a loading control. SLC1A5 levels, normalized to the relative actin levels, are reported as fold change of parental cells. (**C**) Expression of NANOG, SNAT1 and SNAT2 was analyzed by real-time PCR. Data are mean ± S.D. of three independent experiments. (**D**) Dose-dependent effect of GPNA on CD44^+^/CD24^−^ subpopulation. MCF-7 cells were treated for 72 hours with indicated GPNA concentrations. Expression of CD44 and CD24 was analyzed by flow cytometry. Data are mean ± S.D. of three independent experiments. (**E**) GPNA treatment abolishes escape from TIS. Doxorubicin-induced senescent MCF-7 cells were grown in Gln-deprived medium (−Gln), or in complete medium (+Gln) in the absence or in the presence of 1 mM or 2 mM GPNA. Colonies that evaded the senescent growth arrest were stained and counted. Data are mean ± S.D. of two independent experiments. (**F**) GPNA treatment abolishes escape from TIS. Doxorubicin-induced senescent A549 cells were grown in Gln-deprived medium (−Gln), or in complete medium (+Gln) in the absence or in the presence of 0.5 mM or 1 mM GPNA. Colonies that evaded the senescent growth arrest were stained and counted. Data are mean ± S.D. of two independent experiments.

Glutamine uptake from extracellular environment is mediated by different transporters [32]. Among these transporters, SLC1A5/ASCT2 has been shown to be overexpressed in several human malignancies and was associated with low survival [33–35]. In order to confirm enhanced glutamine metabolism in cells that evade TIS, we analyzed the expression of SLC1A5 in MCF-7 escaped clones. As shown in Figure 5A, all escaped clones analyzed showed a significant upregulation of the glutamine transporter, as compared to parental MCF-7 cells. Three clones were selected for further analyses. Increased expression of SLC1A5 was confirmed at protein level in these clones (Figure 5B). Furthermore, compared to parental cells, escaped clones also overexpressed the Na^+^-dependent carrier SNAT1, whereas SNAT2 levels were significantly elevated in just 2 out of three clones (Figure 5C). As expected, escaped clones also overexpressed the stem cell marker Nanog (Figure 5C). Finally, we investigated the effects of L-γ-glutamyl-p-nitroanilide (GPNA), a widely used SLC1A5 inhibitor [33, 35], on MCF-7 CD44/CD24 expression profile. As shown in Figure 5D, GPNA treatment significantly reduced the percentage of the CD44^+^/CD24^−/low^ cells. On the whole, these results further indicate that the CD44^+^/CD24^−/low^ CSC subset involved in TIS evasion in MCF-7 cells is glutamine-dependent and that the glutamine transporter SLC1A5 plays an important role in these cells. Importantly, treatment with GPNA dose-dependently inhibited TIS escape in both MCF-7 and A549 cells (Figure 5E, 5F).

As previously stated, both MCF-7 and A549 cells have a wild-type p53 gene, but TIS can be also induced in cancer cells lacking functional p53 [6], and p53 has been recently shown to play a critical role in adaptation to glutamine deprivation [36, 37]. In order to assess a potential role of p53 in modulating glutamine dependency of escaped cells, we analyzed MDA-MB-231 breast cancer cells, expressing mutant p53 (R280K), which we previously showed to undergo TIS [21]. In addition, we analyzed two murine tumor cell lines: ovarian cancer ID8 cells, which retains wild-type p53 [38] and mammary carcinoma TS/A cells that carries mutant p53 (R270H) [39].

**Figure 6:**
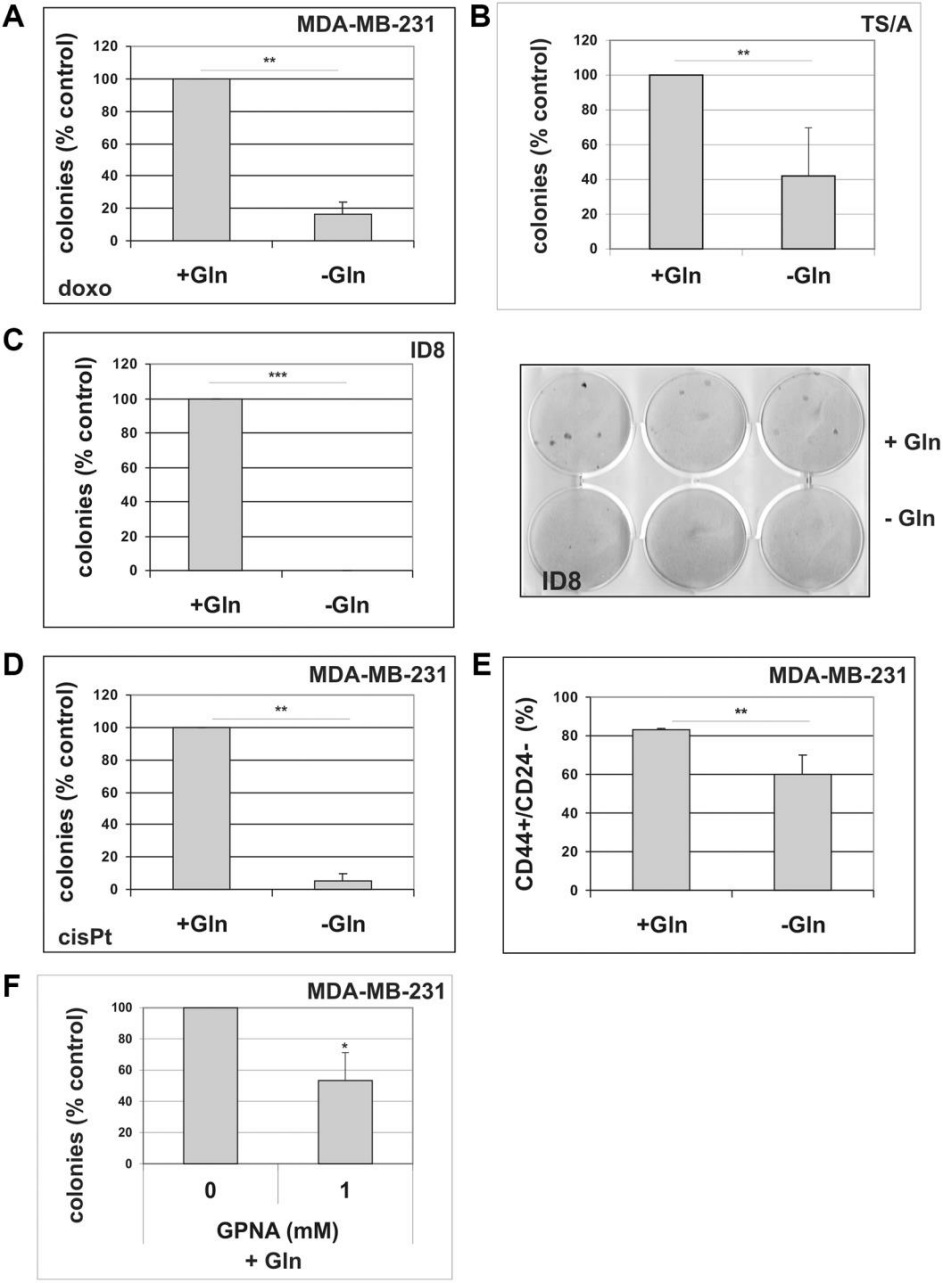
**The effect of glutamine deprivation on TIS escape.** (**A**) Doxorubicin-induced senescent MDA-MB-231 cells were grown in the presence or in the absence of glutamine. Colonies that evaded the senescent growth arrest were counted. Data are mean ± S.D. of three independent experiments. (**B**) Doxorubicin-induced senescent TS/A cells were grown in the presence or in the absence of glutamine. Colonies that evaded the senescent growth arrest were stained and counted. Data are mean ± S.D. of three independent experiments. (**C**) Doxorubicin-induced senescent ID8 cells were grown in the presence or in the absence of glutamine. A representative image of a colony escape assay in ID8 cells is shown. Colonies that evaded the senescent growth arrest were stained and counted. Data are mean ± S.D. of three independent experiments. (**D**) CisPt-induced senescent MDA-MB-231 cells were grown in the presence or in the absence of glutamine. Colonies that evaded the senescent growth arrest were counted. Data are mean ± S.D. of three independent experiments. (**E**) Effect of glutamine on CD44^+^/CD24^−^ subpopulation. MDA-MB-231 cells were grown for 48 hours in the presence or in the absence of glutamine. Expression of CD44 and CD24 was analyzed by flow cytometry. Data are mean ± S.D. of three independent experiments. (**F**) GPNA treatment abolishes escape from TIS. Doxorubicin-induced senescent MDA-MB-231 cells were grown in complete medium (+Gln), in the absence or in the presence of 1 mM GPNA. Colonies that evaded the senescent growth arrest were stained and counted. Data are mean ± S.D. of two independent experiments.

As illustrated in Supplementary Figure 6, MDA-MB-231, ID8 and TS/A cells treated with sublethal concentrations of doxorubicin exhibited a senescent morphology, positive staining for SA-β-gal (Supplementary Figure 6A), upregulation of p21CIP1 and accumulation of hypophosphorylated pRb (Supplementary Figure 6B). All cells underwent a durable growth arrest (Supplementary Figure 6C), after which clones of cell that escape from TIS emerged. We induced TIS in these cells and, after drug removal, maintained them in either complete medium or in Gln-deprived medium. As shown in Figure 6A–6C, glutamine deprivation reduced escape from senescence in all cell lines, with cell line-specific differences not correlated to p53 status. Similar results were obtained after inducing TIS by treatment with the chemotherapeutic cisplatin in MDA-MB-231 cells (Figure 6D). We also analyzed the effect of glutamine deprivation on CD44/CD24 profile in MDA-MB-231 cells. Although MDA-MB-231 line shows over 75% cells with a CD44^+^/CD24^−/low^ phenotype, glutamine-deprivation induced a significant reduction of stem cells subpopulation (Figure 6E and Supplementary Figure 4B). Notably, treatment with SLC1A5 inhibitor GPNA significantly inhibited TIS escape in MDA-MB-231 cells (Figure 6F).

**Figure 7:**
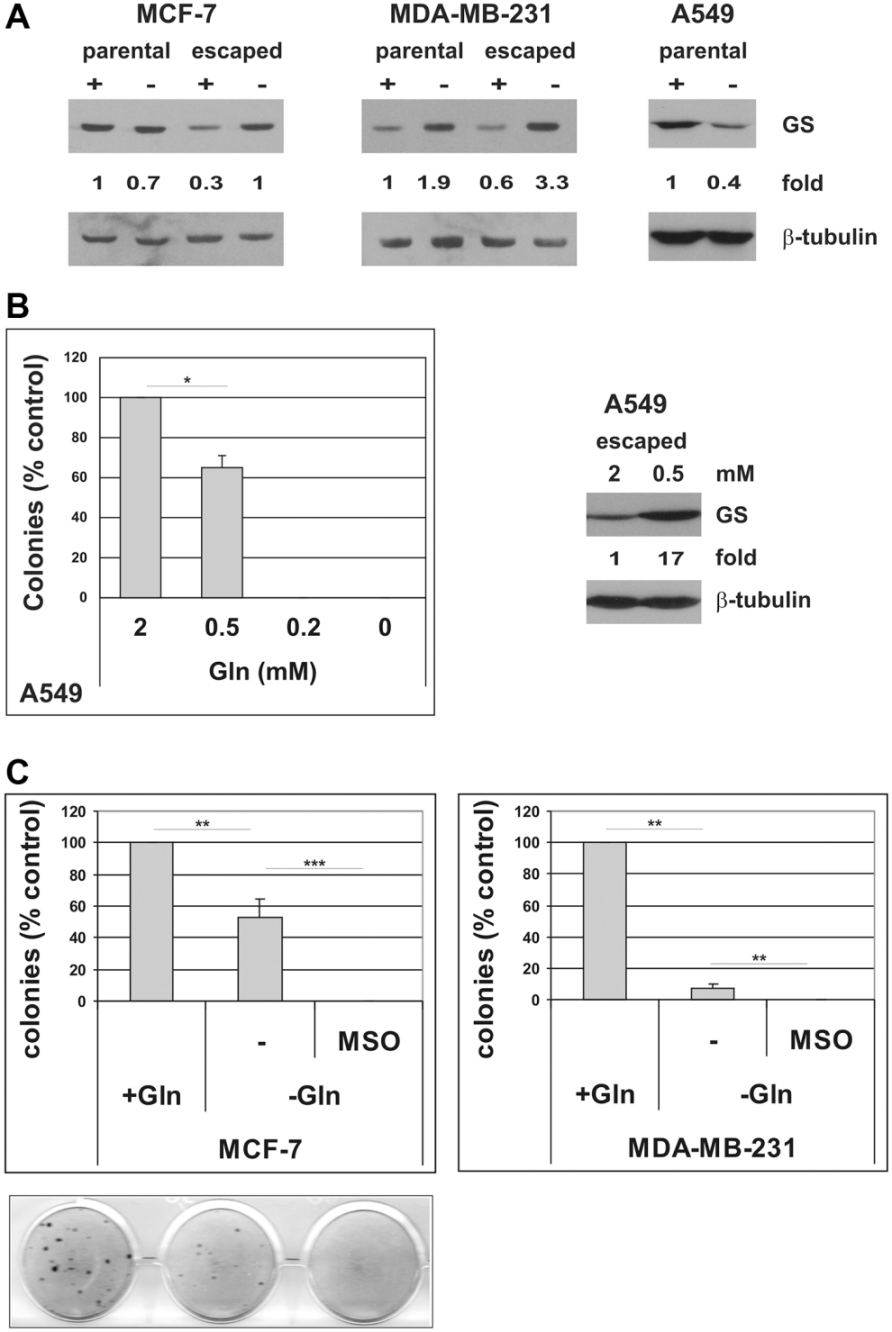
**Induction of GS mediates resistance to glutamine deprivation in cells that escape from TIS.** (**A**) Expression of GS protein was analyzed in parental MCF-7, MDA-MB-231 and A549 cells, grown in the presence or in the absence of glutamine for 72 hours, and in escaped clones arisen in the presence or in the absence of glutamine. Filters were stripped and reprobed with anti-β-tubulin antibodies as a loading control. GS levels, normalized to the relative β-tubulin levels, are reported as fold change of Gln-supplemented parental cells. (**B**) Left panel: doxorubicin-induced senescent A549 cells were grown in media with different glutamine concentrations. Colonies that evaded the senescent growth arrest were stained and counted. Data are mean ± S.D. of two independent experiments. Right panel: expression of GS protein was analyzed in A549 escaped clones arisen in the presence of 2 mM or 0.5 mM glutamine. Filters were stripped and reprobed with anti-β-tubulin antibodies as a loading control. GS levels, normalized to the relative β-tubulin levels, are reported as fold change of Gln-supplemented sample. (**C**) Inhibition of GS with MSO abolishes escape from TIS in Gln-deprived conditions. Doxorubicin-induced senescent MCF-7 and MDA-MB-231 cells were grown in complete medium (+Gln) or in Gln-deprived medium (−Gln), in the presence or in the absence of 2 mM MSO. Colonies that evaded the senescent growth arrest were stained and counted. Data are mean ± S.D. of three independent experiments.

As shown, Gln-deprivation differently affected evasion from TIS depending on the cell line: Gln withdrawal completely abolished escape from TIS in A549 and ID8 cells, while a number of escaped clones could still be detected in MCF-7, MDA-MB-231 and TS/A cell lines. Glutamine independence in cancer cells has been previously associated to increased expression of glutamine synthetase (GS), a cytosolic enzyme that catalyzes the *de novo* synthesis of glutamine from ammonia and glutamate [40–42]. In order to investigate potential mechanisms of resistance that enable escape from TIS in Gln-deprived conditions, we induced senescence in MCF-7 and MDA-MB-231 cells and, after drug removal, incubated the cells in either complete medium or in Gln-deprived medium. When escaped clones emerged, we pooled the clones arisen in the presence or in the absence of glutamine and compared GS protein expression. We also analyzed the expression of GS in the parental cell lines that were Gln-deprived for 72 h. Since, as previously shown, no clones emerge in the absence of glutamine in A549 cells, GS levels were analyzed only in the parental A549 cells with or without Gln for 72 h. As shown in Figure 7A, parental cells showed different responses to Gln-deprivation: MCF-7 showed no alteration in GS protein level, MDA-MB-231 cells upregulated GS and A549 cells downregulated GS in response to glutamine starvation. It may be worth noting that at 72 h Gln-deprivation all parental cell lines analyzed showed significant inhibition of cell proliferation (Figure 2A and Supplementary Figure 7). In contrast, clones of cells that escaped from TIS in Gln-deprived conditions homogeneously showed increased levels of GS, as compared to clones escaped from TIS in presence of glutamine (Figure 7A, and data not shown). We also noticed that the MCF-7 clones that evaded TIS in the presence of glutamine have a lower level of GS as compared to parental cells, which may be related to a more undifferentiated, stem-like phenotype of escaped clones (see discussion). Next, we sought to explore whether GS could also play a role in TIS escape in A549 cells. Since in A549 cells no clones evade TIS in the absence of glutamine, we compared GS levels in clones arisen in the presence of 2 mM glutamine or in presence of the minimal Gln concentration required for escape, i.e. 0.5 mM glutamine (Figure 7B and data not shown). Interestingly, clones of A549 cells that escaped from TIS in Gln-limited conditions showed increased levels of GS, as compared to clones escaped from TIS in presence of 2 mM glutamine (Figure 7B). These data strongly suggest that induction of GS mediates resistance to glutamine deprivation/limitation in cells that escape from TIS. In order to substantiate this role of GS, we induced TIS in MCF-7 and MDA-MB-231 cells and maintained them in either complete medium or in Gln-deprived medium, in the presence or in the absence of 2 mM L-methionine-sulfoximine (MSO), a GS irreversible inhibitor [43]. As shown in Figure 7C, inhibition of GS hampered evasion from TIS in Gln-deprived conditions. On the whole, these data indicate that glutamine promotes escape of CSC from TIS and that increased GS can render CSC resistant to glutamine limitation.

**Figure 8:**
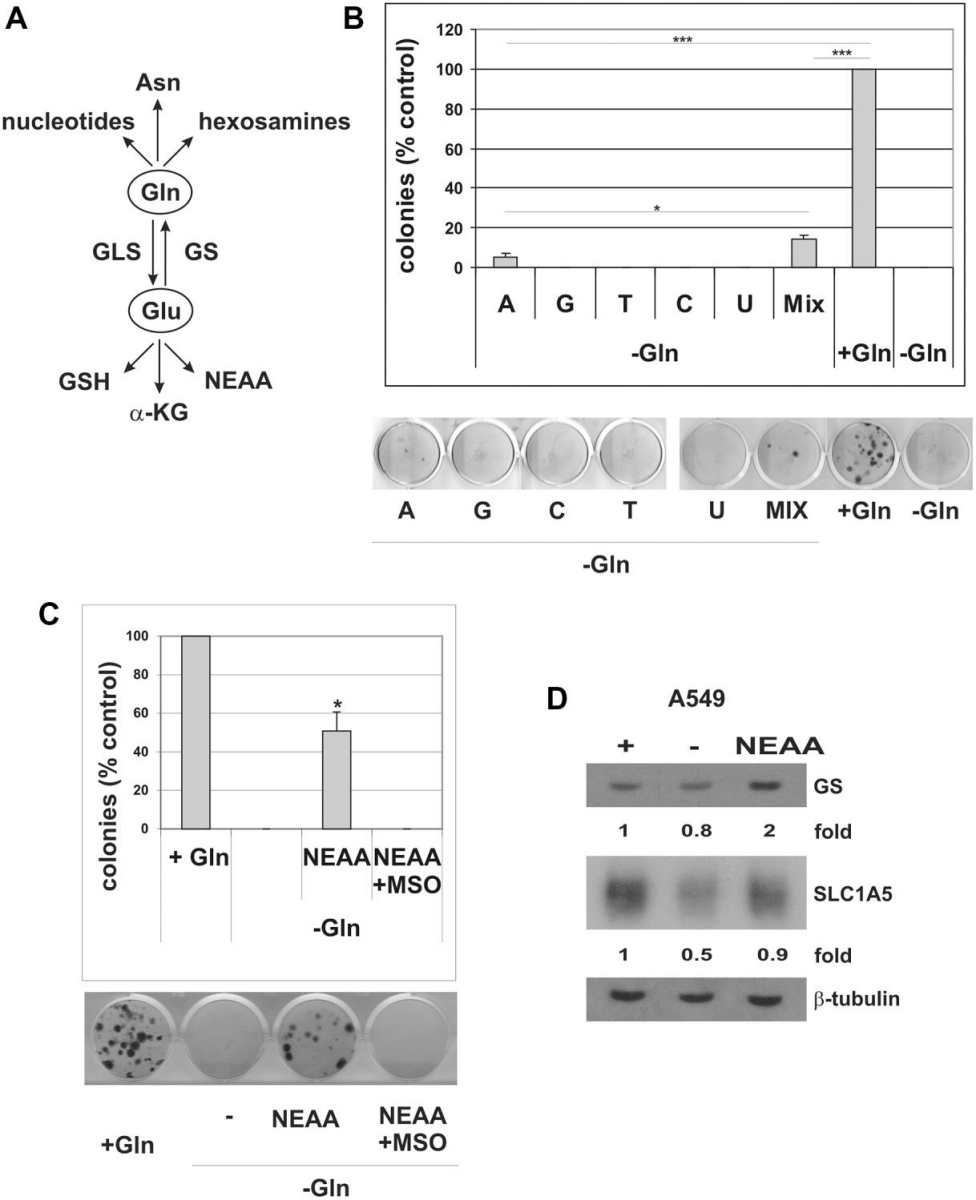
**Nucleosides and NEAA restore escape from TIS in Gln-deprived conditions.** (**A**) Schematic overview of glutamine usage in cancer cells. Gln glutamine, Glu glutamate, Asn asparagine, GSH glutathione, α-KG alpha-ketoglutaric acid, NEAA non-essential amino acids, GLS glutaminase, GS Glutamine synthetase. (**B**) Doxorubicin-induced senescent A549 cells were grown with (+Gln) or without (−Gln) glutamine in medium containing 0.1 mM each adenosine, guanosine, cytidine, thymidine, uridine, or in combination (Mix, 0.1 mM each). Colonies that evaded the senescent growth arrest were stained and counted. The data shown here represent three experiments exhibiting similar effects. A representative image of the colony escape assay is shown. (**C**) Doxorubicin-induced senescent A549 cells were grown with (+Gln), or without (−Gln) glutamine plus NEAA, in the presence or in the absence of 2 mM MSO. Colonies that evaded the senescent growth arrest were stained and counted. The data shown here represent three experiments exhibiting similar effects. A representative image of the colony escape assay is shown. (**D**) Expression of GS and SLC1A5 proteins was analyzed in parental A549 cells, grown with (+) or without (−) glutamine or without glutamine plus NEAA (NEAA) for 72 hours. Filters were stripped and reprobed with anti-β-tubulin antibodies as a loading control. GS and SLC1A5 levels, normalized to the relative β-tubulin levels, are reported as fold change of Gln-supplemented sample.

Cancer cells increase glutamine uptake to supply various biochemical pathways (Figure 8A). On one side, glutamine can be converted to glutamate to provide intermediates for the TCA cycle (anaplerotic flux), for non-essential amino acid (NEAA) biosynthesis, and to support glutathione biosynthesis, thereby controlling the cell redox state [44, 45]. Conversely, glutamine may act as nitrogen donor for biosynthetic pathways, for the synthesis of nucleotides, hexosamines, and asparagine [46]. In order to investigate glutamine utilization in cells that escape from senescence, we released A549 TIS cells in Gln-deprived medium and performed rescue experiments with different glutamine-derived metabolites. It is important to note that A549 cells in glutamine-deprived conditions are unable to upregulate GS, thus excluding confounding results due to resistance mechanisms. As shown in Supplementary Figure 8, supplementation with the glutathione precursor NAC did not rescue Gln-dependent escape. An additional commonly used antioxidant, 2-mercaptoethanol (2ME) also failed to restore Gln-dependent escape (Supplementary Figure 8A). Supplementation with dimethyl-2-oxoglutarate (DM-αKG), a cell-permeable analog of α-ketoglutaric acid, did not rescue TIS escape in the absence of glutamine (Supplementary Figure 8B). Finally, supplementation with either 2 mM asparagine or 2 mM glutamate did not rescue TIS escape (Supplementary Figure 8C). In contrast, the addition of cell-permeable nucleosides partially restored the ability of the cells to escape from TIS under glutamine-limited conditions (Figure 8B). Single nucleosides were ineffective, except for adenosine that restored evasion from TIS but less efficiently than nucleosides mixture. We also observed that escaped clones rescued by nucleosides supplementation were smaller than clones escaped in presence of glutamine. Finally, the addition of a mixture of non-essential amino acids (NEAA: L-alanine, L-asparagine, L-aspartic acid, L-glycine, L-serine, L-proline and L-glutamic acid) greatly restored escape from TIS in glutamine-deprived conditions (Figure 8C). NEAA-rescued clones were found to overexpress SLC1A5 (Figure 8D), which, apart from glutamine, is also involved in transporting other neutral amino acids such as serine, asparagine and alanine [47]. Rather unexpectedly, however, NEAA-rescued clones were also found to overexpress GS (Figure 8D), and the NEAA-dependent rescue was completely abrogated by MSO treatment (Figure 8C). Similar results were obtained with MDA-MB-231 cells (Supplementary Figure 8D). These data indicate that NEAA-dependent rescue of TIS escape requires the activity of GS and subsequent glutamine synthesis. A schematic summary of the proposed role of GS in TIS evasion in cancer is shown in Supplementary Figure 9.

## DISCUSSION

Therapy-induced senescence is recognized as a major cellular response to cytotoxic chemotherapies and radiotherapies [1, 2], as well as to targeted cancer therapy (reviewed in [48]). Both positive and negative effects of TIS have been described. First, while induction of apoptosis in tumor cells requires high levels of damage, TIS is induced by low dose chemo- and radiotherapy, possibly reducing side effects in patients [3]. Second, senescent tumor cells develop a secretory phenotype or SASP, and secrete cytokines and growth factors [5] that reinforce the senescent growth arrest and activate the anti-tumor immune response [49]. On the other hand, the SASP also mediates several pro-tumorigenic effects of TIS. For instance, SASP can promote adverse effects of chemotherapy and tumor relapse [50].

Another concern about TIS is the ability of cancer cells to evade the senescence cell cycle arrest, resuming proliferation after a long period of time [6, 7], likely causing tumor recurrence after chemo- or radiotherapy [8, 51]. Notably, only a small subset of cells characterized by an aggressive, stem cell-like phenotype is endowed with the ability to escape from TIS [14, 15, 16]. Although the mechanisms responsible for senescence reversal in TIS are not fully understood, it has been demonstrated that induction of TIS by itself results in a genetic reprogramming that promotes stemness and malignancy [16].

It is known that CSC show distinct metabolic features relative to bulk tumor cells [17, 18], yet the metabolic requirements of CSC that escape from TIS have not been fully investigated. In this study we show that cancer cells rely on glutamine for TIS escape, which thus can be simply modulated by varying the concentrations of glutamine in the medium. Notably, also the percentage of CD44^+^/CD24^−/low^ cells can be modulated in breast tumor cell lines by altering glutamine concentration in culture medium. These results, together with our observation that escaped clones isolated from MCF-7 TIS cells are enriched in CD44^+^/CD24^−/low^ cells, confirm previous reports showing a functional link between escape from TIS and cancer stemness [14, 16] and reveals a metabolic dependency of CSC that evade TIS. In line with a central role of glutamine metabolism in cells that escape from TIS, we also demonstrate that MCF-7 escaped clones overexpress the glutamine transporters SLC1A5 and SNAT1. Accordingly, treatment with GPNA, a SLC1A5 and SNAT family inhibitor [52], dose-dependently reduces the percentage of the CD44^+^/CD24^−/low^ cells in MCF-7 line, and inhibits TIS evasion in all cell lines analyzed. However, it is important to note that GPNA has also been demonstrated to inhibit the system L transporters LAT1 and LAT2 and to hinder the uptake of essential amino acids, and leucine in particular [53]. Furthermore, the γ-glutamyltransferase (GGT)-dependent hydrolysis of GPNA releases p-nitroaniline (PNA), which has been shown to affect cell viability [54]. Hence, the role of amino acids transporters in modulating escape from TIS in cancer cells will need further investigations.

We further substantiate the glutamine-dependency of cancer-initiating cells by showing that glutamine deprivation selectively inhibits holoclone formation. Generation of holoclones in monoclonal cultivation represents a new *in vitro* method for studying cancer stem cells [55]. It has been demonstrated that when cancer cells are cloned, three types of colony grow (holoclones, meroclones and paraclones), which reproduce the vertical hierarchy of CSC compartment [56]. Notably, stem cell surface markers, genes and microRNAs are specifically expressed in holoclones [30, 55]. In our experiments, monoclonal cultivation of MCF-7 cells in glutamine-deprived conditions hampers the formation of holoclones, while increases the percentage of paraclones. Furthermore, increasing glutamine concentrations from 2 mM to 4 mM causes a significant increase in holoclones formation. Interestingly, while the relative frequency of holoclones (indicative of stemness) changes proportionally to glutamine concentration, the size of colonies (reflecting cell proliferation) is not significantly affected. This observation strongly suggests that the observed effects of glutamine deprivation or enrichment reflect an ability of glutamine to modulate cancer cell stemness and not merely cell proliferation. Since meroclones and paraclones do not retain the self-renewal ability of holoclones [57], these results suggest that glutamine may be required for CSC self-renewal. Further investigations will be required to confirm this possibility.

It is well known that glycolysis plays a key role in cancer [58]. Tumor cells exhibit increase in glucose uptake, and high levels of glucose promote tumor cell proliferation, motility and chemoresistance [59, 60]. By using low glucose-containing medium or 2DG to inhibit glucose metabolism, we show that glucose restriction is well tolerated by parental cells and does not affect escape from TIS. Similarly, pharmacological inhibition of glycolysis by 2DG does not impact evasion from TIS, even though the concentration of 2DG used in these experiments (5 mM) reduced by more than 50% the proliferation of MCF-7 and A549 cells after 72 hours (Figure 2A). Hence, it appears that cells that escape the TIS growth arrest can exhibit distinct susceptibility to metabolic perturbations, as compared to the parental cells. Different mechanisms of adaptation to glycolysis inhibition have been described in cancer (reviewed in [61]). For instance, glycolytic suppression induced metabolic reprogramming toward mitochondrial oxidative phosphorylation in different tumor types [62, 63]. An increased expression of glycolytic enzymes, mediated by Hypoxia-inducible factor (HIF), led to increased resistance to 2DG [64]. Furthermore, treatment with 2DG activated several pro-survival pathways through IGF1R [65]. The metabolic adaptation(s) occurring in escaped clones, which confer resistance to 2DG, will need further investigations. Alternatively, increased resistance to glycolysis inhibition might reflect a general metabolic rewiring of cancer cells experiencing evasion from TIS.

The existence of escape-specific metabolic adaptations is further supported by the uniform upregulation of glutamine synthetase shared by all clones that evade TIS in Gln-deprived or Gln-limited conditions, as compared to clones that escape in presence of glutamine. Analyses of GS protein levels in parental tumor cell lines grown in glutamine-deprived or glutamine-supplemented conditions show marked variations between parental cells, with increased GS levels in MDA-MB-231 cells, no alteration in MCF-7 cells and decreased levels in A549 cells in response to glutamine deprivation. In contrast, glutamine limitation consistently induces increased GS expression in clones that escape from TIS. Accordingly, we show that pharmacological inhibition of GS with MSO completely blunts evasion from TIS in glutamine-deprived conditions in both MCF-7 and MDA-MB-231 cells. These results again underscore the metabolic dependency of cells that evade TIS on glutamine.

Differential expression of GS in different cancer cell lines has been previously reported, and correlated to their dependence on glutamine [45]. Interestingly, luminal-type breast cells were more glutamine-independent than basal-type, due to lineage-specific upregulation of GS [42]. Our data showing different basal expression of GS protein in MCF-7 and MDA-MB-231 cells (Figure 7A), respectively luminal- and basal-type cells, are consistent with these previous findings, but also provide evidence for a differential regulation of GS, which is induced in MDA-MB-231 cells in response to glutamine deprivation, whereas does not change in MCF-7 cells. Hence, these observations suggest an ability of basal-type breast cells to upregulate GS when facing glutamine shortage. In this context, it is interesting to note that escaped clones that emerge from senescent MCF-7 cells in the presence of glutamine show a significantly lower level of GS as compared to parental cells (Figure 7A). Since clones that escape from TIS acquire a stem-like phenotype, it is plausible to hypothesize that reduced GS protein expression reflects an undifferentiated, “non-luminal” phenotype. In contrast, GS protein levels decrease in A549 under glutamine-depleted conditions, likely due to general suppression of protein synthesis [45, 46, 66]. The inability of A549 cells to upregulate GS may render these cells especially vulnerable to glutamine withdrawal, severely impairing the proliferation of parental cells (Supplementary Figure 1B), and completely preventing escape from TIS (Figure 2B).

GS has been demonstrated to be required for adaptation to glutamine deprivation in different tumor types. For instance, GS was required for sarcoma adaptation and growth under glutamine deprivation [40]. The reduction of cell survival induced by glutamine depletion was inversely correlated with the expression level of GS in ovarian cancer cells [67]. GS activity was sufficient to maintain glioblastoma proliferation in the absence of exogenous glutamine [41]. Notably, these authors showed that when primary glioblastoma cells were maintained in a stem-like state, GS expression was dramatically increased, again linking glutamine metabolism to cancer stemness. In our study, we show that expression of GS confers the ability to evade TIS in the absence of glutamine, and that blocking GS with MSO prevents TIS escape in glutamine-deprived conditions. On the whole, these data demonstrate a central role of GS in mediating resistance to glutamine deprivation in CSC that escape from TIS.

Increased glutamine uptake promotes cancer cell growth by supplying different biochemical pathways. First, glutamine can serve as carbon source via conversion to glutamate, and then to α-ketoglutarate, which enters the TCA cycle via glutaminolysis for energy production (anaplerotic flux) [44]. Glutamine-derived glutamate also contributes to the biosynthesis of NEAA and glutathione, thus supporting cellular antioxidant defenses [68]. On the other hand, glutamine may serve as nitrogen donor for the biosynthesis of nucleotides, glucosamine and asparagine [44, 66]. Here, our rescue experiments indicate that in cells that evade TIS glutamine is not used for fueling the TCA cycle, since supplementation with either glutamate or DM-αKG fails to restore escape under glutamine-deprived conditions. This interpretation is also supported by the fact that the first rate-limiting enzyme in glutaminolysis, glutaminase (GLS), converts glutamine to glutamate, whereas escaped clones upregulate GS which catalyzes the reverse reaction (Figure 8A). The observation that glutamine requirement in cells that escape from TIS is independent from anaplerotic TCA usage also reinforces an important role of ASCT2 as glutamine transporter in these cells, since it has been demonstrated that in cancer cells SNAT1 and SNAT2 mediate net glutamine uptake for glutaminolysis, whereas ASCT2 is required for optimal growth at low glutamine concentrations [52]. Glutamine-derived glutamate also contributes to maintain redox homeostasis though glutathione synthesis. However, supplementation of glutamine-deprived TIS cells with the glutathione precursor NAC or with the free radical scavenger beta-mercaptoethanol, fails to rescue evasion from TIS.

It has been demonstrated that exogenous asparagine can rescue growth and survival of glutamine-deprived cells by maintaining protein synthesis [69], but asparagine supplementation does not restore escape from TIS in our cell system.

On the other hand, we show that the combined addition of nucleosides, as well as simple adenosine addition, partially restores evasion from TIS in glutamine-deprived A549 cells. The importance of glutamine in sustaining *de novo* nucleotide synthesis to support rapid proliferation in cancer cells has been previously demonstrated in different cancer types, and, notably, has been linked to GS upregulation [41, 70]. However, in line with previous studies [41, 71], we observe that nucleosides supplementation only partially rescued the ability of cancer cells to evade the TIS growth arrest in the absence of glutamine. Although limited, the ability of exogenous nucleosides to restore TIS escape support a role of glutamine as nitrogen donor, but also suggest a critical involvement of glutamine in other pathways besides nucleotide synthesis.

Finally, we show that the addition of NEAA greatly restores escape from TIS in glutamine-deprived conditions. However, clones that escape from TIS in the presence of NEAA overexpress GS, and inhibition of GS activity by MSO completely abolish NEAA-dependent rescue. These data strongly suggest that NEAA supplementation restores TIS escape not by providing biosynthetic intermediates downstream of glutamine, but by enabling GS expression and subsequent glutamine biosynthesis. We hypothesize that this effect is related to NEAA-dependent recovery of protein synthesis. These findings again underscore a critical role of GS and glutamine in cells that evade TIS. Since glutamine role in cancer cells extends beyond metabolism [72] non-metabolic functions of glutamine in cells that evade TIS are under investigation in our lab.

As a whole, these data reveal a metabolic vulnerability of CSC that recover proliferation after exposure to anticancer therapies, and suggest that pharmacologic inhibition of GS could be exploited to prevent tumor recurrence after chemotherapy.

## METHODS

### Cell culture and biological reagents

A549, MCF-7 and MDA-MB-231 cells were obtained from American Type Culture Collection. ID8 cells were kindly provided by M. D’Incalci (IRCCS Istituto di Ricerche Farmacologiche Mario Negri, Milan, Italy).

All media were supplemented with 10% fetal bovine serum (FBS). DMEM without L-glutamine was purchased from Immunological Sciences (Società Italiana Chimici, Italy). DMEM low glucose (1 g/L glucose), L-glutamine 200 mM solution and Non-essential Amino Acid solution were purchased from Sigma-Aldrich (Sigma-Aldrich, Milan, Italy). L-γ-glutamyl-p-nitroanilide (GPNA) was purchased from Sigma-Aldrich (Sigma-Aldrich, Milan, Italy).

All chemicals were purchased from Sigma-Aldrich (Sigma-Aldrich, Milan, Italy). Doxorubicin hydrochloride was dissolved in sterile water; cisplatin (cis-diammineplatinum (II) dichloride) was dissolved in PBS; L-NAC was dissolved in sterile water; MSO was dissolved in culture medium; 2-deoxy-D-glucose (2DG) was dissolved in culture medium; nucleosides were dissolved in sterile water or in culture medium. GPNA was dissolved in DMSO.

### Induction of premature senescence and SA-β-gal staining

Unless otherwise stated, senescence was induced by treating cells with the DNA-damaging agent doxorubicin (200 nM for MCF-7, MDA-MB-231, ID8 cells and 600 nM for A549 and TS/A cells) for 72 h. Where indicated, cisplatin (10 μM for 24 h) was used to induce senescence in MCF-7 and MDA-MB-231 cells. Staining for SA-β-gal was performed as previously described [73].

### Cell viability

Cell viability was determined by trypan blue exclusion assay. 4 × 10^3^ proliferating cells were seeded in triplicate into 96-well plates. 16 h later, cells were treated as indicated. Cell viability was estimated 72 h after treatments.

### Escape from senescence

Senescent A549 (8 × 10^4^), MCF-7 (1.2 × 10^5^) and MDA-MB-231 (1.2 × 10^5^) cells were plated in triplicate in a 12-well plate. After 24 h cells were treated as indicated and incubated under standard conditions. Media were changed and treatments were repeated twice a week. Fifteen to sixty days after drug washout colonies that escaped senescence were stained with 1% Methylene Blue (Sigma-Aldrich, Milan, Italy) in 50% (v/v) ethanol. Colonies ≥50 cells were counted. When isolated or pooled for analyses, colonies were counted at light microscope, placing a grid underneath the dishes. MDA-MB-231 cells form non-compact colonies that were always counted using a grid. Escaped clones were isolated using cloning rings.

### Clonal heterogeneity analysis

Cells were seeded in triplicate at 200 cells/cm^2^ in complete medium to allow for cell attachment. After 24 h cells were maintained in either Gln-deprived medium or in media with different glutamine concentrations. Media was changed twice a week. After 7 to 10 days, colonies from three individual clonal cultures were counted and classified as holoclone, meroclone, and paraclone. More than 150 colonies were counted by two independent investigators. In order to estimate average holoclone dimensions, cells were washed and phase-contrast microscopy pictures were taken. Area and measure tools (ImageJ software) were used to estimate colony sizes (as represented in Supplementary Figure 5), and colony areas were expressed in square pixels. For each experimental condition, more than 50 holoclones were analyzed.

### mRNA Quantification by real-time RT-PCR

Real-time RT-PCR was carried out with cDNAs reverse-transcribed from total RNA by using cDNA Synthesis SuperMix (ABM Applied Biological Materials Inc., Bellingham, WA, USA) and amplified using SensiFAST™ SYBR No-ROX Kit (Bioline, London, UK). Relative mRNA quantitation was performed using the Bio-Rad CFX Manager software.

The primers were:

SLC1A5: 5′-CCTTTCGCTCATACTCTACCAC-3′; 5′-AAACACTACCAAGCCCAGG -3′.

SNAT1: 5′-CATTATGGGCAGTGGGATTTTG-3′; 5′-TGCAGCCTGTTTCTTTTGAAC-3′.

SNAT2: 5′-CCAGGCATTAACGAACATTGAAG-3′; 5′-CACCAATGACACCAACAGAAC-3′.

CYPA: 5′-CCGAGGAAAACCGTGTACTATTAG-3′; 5′TGCTGTCTTTGGGACCTTG-3′.

### Flow cytometry

Cell surface expression of breast stem cell markers CD44 and CD24 was analyzed by flow cytometry. Breast cells were harvested with phosphate-buffered saline (PBS), 2 mM ethylenediaminetetraacetic acid (EDTA) and washed with PBS supplemented with 1% fetal bovine serum. Cells were resuspended in PBS, and stained with anti-CD44 (FITC-conjugated; cat. no. 555478, BD Biosciences San Jose, CA, USA) and anti-CD24 (PE-conjugated; cat. no. 555428, BD Biosciences) at 4°C in the dark for 30 min, according to the manufacturer’s instructions.

For cell cycle analysis, cells were fixed with 70% ethanol in PBS. Cells were washed with PBS, resuspended in PBS, 40 μg/ml propidium iodide (Sigma-Aldrich, Milan, Italy), 50 μg/ml RNase DNase-free (Sigma-Aldrich, Milan, Italy) and incubated in the dark at room temperature for 20 min.

Flow cytometry analyses were performed on a BD Accuri™ C6 Flow Cytometer (BD Biosciences).

### Western blot analysis

Total cell proteins preparations and Western blots analyses were performed as previously reported [19]. The anti-Glutamine synthetase (GS) antibody was from Sigma (Sigma-Aldrich, Milan, Italy); anti-p21 (C-19), actin (I-19) and β-Tubulin (G-8) antibodies were from Santa Cruz Biotechnology (Santa Cruz, CA, USA). The anti-pRb antibody was from BD Biosciences (San Jose, CA, USA).

### LC−MS analysis

A549 and MCF-7 cells were grown for 72 hours in the presence or in the absence of 2 mM glutamine. The cell monolayers were rinsed twice with PBS, scraped in distilled water, and stored at –80°C for further analysis. The samples were vortex-mixed, kept on ice for 20 min, and centrifuged again at 10 000 g, at 4°C for 10 min. The collected supernatants were dried-up in a speedvac system operated at room temperature. Dried supernatants were reconstituted with 125 μL of methanol/acetonitrile/water (50:25:25). Metabolites extracted from *in vitro* growing cells were analyzed using an ACQUITY UPLC system online coupled to a Synapt G2-Si QTOF-MS (Waters Corporation, Milford, MA, USA) in positive mode in the following settings: reverse-phase ACQUITY UPLC CSH C18 (1.7 μm, 100 × 2.1 mm^2^ ) column (Waters), 0.3 mL/min flow rate, mobile phases composed of acetonitrile/H20 (60:40) containing 0.1% formic acid and 10 mM ammonium formate (Phase A), and isopropanol/acetonitrile (90:10) containing 0.1% formic acid and 10 mM ammonium formate (Phase B). Glutamine peak detection and quantitation was achieved upon fitting experimental data with internal standard and calibration curves.

### Statistical analysis

Statistical significance was determined using unpaired Student’s *t*-test or one-sample *t*-test (for normalized data). *p*-values ≤ 0.05 were considered statistically significant. In all the manuscript: ^*^p ≤ 0.05; ^**^p ≤ 0.005; ^***^p ≤ 0.0001.

## Supplementary Materials

Supplementary Figures

## References

[1] te Poele RH, Okorokov AL, Jardine L, Cummings J, Joel SP. DNA damage is able to induce senescence in tumor cells in vitro and in vivo. Cancer Res. 2002; 62:1876–83. 11912168

[2] Mirzayans R, Scott A, Cameron M, Murray D. Induction of accelerated senescence by gamma radiation in human solid tumor-derived cell lines expressing wild-type TP53. Radiat Res. 2005; 163:53–62. 10.1667/rr3280 15606307

[3] Ewald JA, Desotelle JA, Wilding G, Jarrard DF. Therapy-induced senescence in cancer. J Natl Cancer Inst. 2010; 102:1536–46. 10.1093/jnci/djq364 20858887 PMC2957429

[4] Demaria M, O'Leary MN, Chang J, Shao L, Liu S, Alimirah F, Koenig K, Le C, Mitin N, Deal AM, Alston S, Academia EC, Kilmarx S, et al. Cellular Senescence Promotes Adverse Effects of Chemotherapy and Cancer Relapse. Cancer Discov. 2017; 7:165–76. 10.1158/2159-8290.CD-16-0241 27979832 PMC5296251

[5] Coppé JP, Patil CK, Rodier F, Sun Y, Muñoz DP, Goldstein J, Nelson PS, Desprez PY, Campisi J. Senescence-associated secretory phenotypes reveal cell-nonautonomous functions of oncogenic RAS and the p53 tumor suppressor. PLoS Biol. 2008; 6:2853–68. 10.1371/journal.pbio.0060301 19053174 PMC2592359

[6] Roberson RS, Kussick SJ, Vallieres E, Chen SY, Wu DY. Escape from therapy-induced accelerated cellular senescence in p53-null lung cancer cells and in human lung cancers. Cancer Res. 2005; 65:2795–803. 10.1158/0008-5472.CAN-04-1270 15805280

[7] Elmore LW, Di X, Dumur C, Holt SE, Gewirtz DA. Evasion of a single-step, chemotherapy-induced senescence in breast cancer cells: implications for treatment response. Clin Cancer Res. 2005; 11:2637–43. 10.1158/1078-0432.CCR-04-1462 15814644

[8] Saleh T, Tyutyunyk-Massey L, Gewirtz DA. Tumor Cell Escape from Therapy-Induced Senescence as a Model of Disease Recurrence after Dormancy. Cancer Res. 2019; 79:1044–46. 10.1158/0008-5472.CAN-18-3437 30803994

[9] Wu PC, Wang Q, Grobman L, Chu E, Wu DY. Accelerated cellular senescence in solid tumor therapy. Exp Oncol. 2012; 34:298–305. 23070015

[10] Supiot S, Shubbar S, Fleshner N, Warde P, Hersey K, Wallace K, Cole H, Sweet J, Tsihlias J, Jewett MA, Klotz L, Bristow RG. A phase I trial of pre-operative radiotherapy for prostate cancer: clinical and translational studies. Radiother Oncol. 2008; 88:53–60. 10.1016/j.radonc.2008.03.019 18423916

[11] Sidi R, Pasello G, Opitz I, Soltermann A, Tutic M, Rehrauer H, Weder W, Stahel RA, Felley-Bosco E. Induction of senescence markers after neo-adjuvant chemotherapy of malignant pleural mesothelioma and association with clinical outcome: an exploratory analysis. Eur J Cancer. 2011; 47:326–32. 10.1016/j.ejca.2010.09.044 21036600

[12] Kim SB, Bozeman RG, Kaisani A, Kim W, Zhang L, Richardson JA, Wright WE, Shay JW. Radiation promotes colorectal cancer initiation and progression by inducing senescence-associated inflammatory responses. Oncogene. 2016; 35:3365–75. 10.1038/onc.2015.395 26477319 PMC4837107

[13] Karimi-Busheri F, Rasouli-Nia A, Mackey JR, Weinfeld M. Senescence evasion by MCF-7 human breast tumor-initiating cells. Breast Cancer Res. 2010; 12:R31. 10.1186/bcr2583 20525204 PMC2917024

[14] Achuthan S, Santhoshkumar TR, Prabhakar J, Nair SA, Pillai MR. Drug-induced senescence generates chemoresistant stemlike cells with low reactive oxygen species. J Biol Chem. 2011; 286:37813–29. 10.1074/jbc.M110.200675 21878644 PMC3199523

[15] Ansieau S, Bastid J, Doreau A, Morel AP, Bouchet BP, Thomas C, Fauvet F, Puisieux I, Doglioni C, Piccinin S, Maestro R, Voeltzel T, Selmi A, et al. Induction of EMT by twist proteins as a collateral effect of tumor-promoting inactivation of premature senescence. Cancer Cell. 2008; 14:79–89. 10.1016/j.ccr.2008.06.005 18598946

[16] Milanovic M, Fan DNY, Belenki D, Däbritz JHM, Zhao Z, Yu Y, Dörr JR, Dimitrova L, Lenze D, Monteiro Barbosa IA, Mendoza-Parra MA, Kanashova T, Metzner M, et al. Senescence-associated reprogramming promotes cancer stemness. Nature. 2018; 553:96–100. 10.1038/nature25167 29258294

[17] Sancho P, Barneda D, Heeschen C. Hallmarks of cancer stem cell metabolism. Br J Cancer. 2016; 114:1305–12. 10.1038/bjc.2016.152 27219018 PMC4984474

[18] Snyder V, Reed-Newman TC, Arnold L, Thomas SM, Anant S. Cancer Stem Cell Metabolism and Potential Therapeutic Targets. Front Oncol. 2018; 8:203. 10.3389/fonc.2018.00203 29922594 PMC5996058

[19] Crescenzi E, Raia Z, Pacifico F, Mellone S, Moscato F, Palumbo G, Leonardi A. Down-regulation of wild-type p53-induced phosphatase 1 (Wip1) plays a critical role in regulating several p53-dependent functions in premature senescent tumor cells. J Biol Chem. 2013; 288:16212–24. 10.1074/jbc.M112.435149 23612976 PMC3675561

[20] Crescenzi E, Pacifico F, Lavorgna A, De Palma R, D'Aiuto E, Palumbo G, Formisano S, Leonardi A. NF-κB-dependent cytokine secretion controls Fas expression on chemotherapy-induced premature senescent tumor cells. Oncogene. 2011; 30:2707–17. 10.1038/onc.2011.1 21278794

[21] Camorani S, Cerchia L, Fedele M, Erba E, D'Incalci M, Crescenzi E. Trabectedin modulates the senescence-associated secretory phenotype and promotes cell death in senescent tumor cells by targeting NF-κB. Oncotarget. 2018; 9:19929–44. 10.18632/oncotarget.24961 29731994 PMC5929437

[22] Yang L, Fang J, Chen J. Tumor cell senescence response produces aggressive variants. Cell Death Discov. 2017; 3:17049. 10.1038/cddiscovery.2017.49 28845296 PMC5563524

[23] Al-Hajj M, Wicha MS, Benito-Hernandez A, Morrison SJ, Clarke MF. Prospective identification of tumorigenic breast cancer cells. Proc Natl Acad Sci U S A. 2003; 100:3983–88. 10.1073/pnas.0530291100 12629218 PMC153034

[24] Vazquez A, Kamphorst JJ, Markert EK, Schug ZT, Tardito S, Gottlieb E. Cancer metabolism at a glance. J Cell Sci. 2016; 129:3367–73. 10.1242/jcs.181016 27635066 PMC6518336

[25] Reckzeh ES, Karageorgis G, Schwalfenberg M, Ceballos J, Nowacki J, Stroet MCM, Binici A, Knauer L, Brand S, Choidas A, Strohmann C, Ziegler S, Waldmann H. Inhibition of Glucose Transporters and Glutaminase Synergistically Impairs Tumor Cell Growth. Cell Chem Biol. 2019; 26:1214–28.e25. 10.1016/j.chembiol.2019.06.005 31303578

[26] Li D, Fu Z, Chen R, Zhao X, Zhou Y, Zeng B, Yu M, Zhou Q, Lin Q, Gao W, Ye H, Zhou J, Li Z, et al. Inhibition of glutamine metabolism counteracts pancreatic cancer stem cell features and sensitizes cells to radiotherapy. Oncotarget. 2015; 6:31151–63. 10.18632/oncotarget.5150 26439804 PMC4741594

[27] Liao J, Liu PP, Hou G, Shao J, Yang J, Liu K, Lu W, Wen S, Hu Y, Huang P. Regulation of stem-like cancer cells by glutamine through β-catenin pathway mediated by redox signaling. Mol Cancer. 2017; 16:51. 10.1186/s12943-017-0623-x 28245869 PMC5331650

[28] Yadav UP, Singh T, Kumar P, Sharma P, Kaur H, Sharma S, Singh S, Kumar S, Mehta K. Metabolic Adaptations in Cancer Stem Cells. Front Oncol. 2020; 10:1010. 10.3389/fonc.2020.01010 32670883 PMC7330710

[29] Barrandon Y, Green H. Three clonal types of keratinocyte with different capacities for multiplication. Proc Natl Acad Sci U S A. 1987; 84:2302–06. 10.1073/pnas.84.8.2302 2436229 PMC304638

[30] Tan L, Sui X, Deng H, Ding M. Holoclone forming cells from pancreatic cancer cells enrich tumor initiating cells and represent a novel model for study of cancer stem cells. PLoS One. 2011; 6:e23383. 10.1371/journal.pone.0023383 21826251 PMC3149653

[31] Tièche CC, Gao Y, Bührer ED, Hobi N, Berezowska SA, Wyler K, Froment L, Weis S, Peng RW, Bruggmann R, Schär P, Amrein MA, Hall SRR, et al. Tumor Initiation Capacity and Therapy Resistance Are Differential Features of EMT-Related Subpopulations in the NSCLC Cell Line A549. Neoplasia. 2019; 21:185–96. 10.1016/j.neo.2018.09.008 30591423 PMC6309124

[32] Pochini L, Scalise M, Galluccio M, Indiveri C. Membrane transporters for the special amino acid glutamine: structure/function relationships and relevance to human health. Front Chem. 2014; 2:61. 10.3389/fchem.2014.00061 25157349 PMC4127817

[33] Hassanein M, Hoeksema MD, Shiota M, Qian J, Harris BK, Chen H, Clark JE, Alborn WE, Eisenberg R, Massion PP. SLC1A5 mediates glutamine transport required for lung cancer cell growth and survival. Clin Cancer Res. 2013; 19:560–70. 10.1158/1078-0432.CCR-12-2334 23213057 PMC3697078

[34] Cormerais Y, Massard PA, Vucetic M, Giuliano S, Tambutté E, Durivault J, Vial V, Endou H, Wempe MF, Parks SK, Pouyssegur J. The glutamine transporter ASCT2 (SLC1A5) promotes tumor growth independently of the amino acid transporter LAT1 (SLC7A5). J Biol Chem. 2018; 293:2877–87. 10.1074/jbc.RA117.001342 29326164 PMC5827425

[35] van Geldermalsen M, Wang Q, Nagarajah R, Marshall AD, Thoeng A, Gao D, Ritchie W, Feng Y, Bailey CG, Deng N, Harvey K, Beith JM, Selinger CI, et al. ASCT2/SLC1A5 controls glutamine uptake and tumour growth in triple-negative basal-like breast cancer. Oncogene. 2016; 35:3201–08. 10.1038/onc.2015.381 26455325 PMC4914826

[36] Lowman XH, Hanse EA, Yang Y, Ishak Gabra MB, Tran TQ, Li H, Kong M. p53 Promotes Cancer Cell Adaptation to Glutamine Deprivation by Upregulating Slc7a3 to Increase Arginine Uptake. Cell Rep. 2019; 26:3051–60.e4. 10.1016/j.celrep.2019.02.037 30865893 PMC6510239

[37] Tajan M, Hock AK, Blagih J, Robertson NA, Labuschagne CF, Kruiswijk F, Humpton TJ, Adams PD, Vousden KH. A Role for p53 in the Adaptation to Glutamine Starvation through the Expression of SLC1A3. Cell Metab. 2018; 28:721–36.e6. 10.1016/j.cmet.2018.07.005 30122553 PMC6224545

[38] Walton J, Blagih J, Ennis D, Leung E, Dowson S, Farquharson M, Tookman LA, Orange C, Athineos D, Mason S, Stevenson D, Blyth K, Strathdee D, et al. CRISPR/Cas9-Mediated Trp53 and Brca2 Knockout to Generate Improved Murine Models of Ovarian High-Grade Serous Carcinoma. Cancer Res. 2016; 76:6118–29. 10.1158/0008-5472.CAN-16-1272 27530326 PMC5802386

[39] Odin L, Favrot M, Poujol D, Michot JP, Moingeon P, Tartaglia J, Puisieux I. Canarypox virus expressing wild type p53 for gene therapy in murine tumors mutated in p53. Cancer Gene Ther. 2001; 8:87–98. 10.1038/sj.cgt.7700279 11263530

[40] Issaq SH, Mendoza A, Fox SD, Helman LJ. Glutamine synthetase is necessary for sarcoma adaptation to glutamine deprivation and tumor growth. Oncogenesis. 2019; 8:20. 10.1038/s41389-019-0129-z 30808861 PMC6391386

[41] Tardito S, Oudin A, Ahmed SU, Fack F, Keunen O, Zheng L, Miletic H, Sakariassen PØ, Weinstock A, Wagner A, Lindsay SL, Hock AK, Barnett SC, et al. Glutamine synthetase activity fuels nucleotide biosynthesis and supports growth of glutamine-restricted glioblastoma. Nat Cell Biol. 2015; 17:1556–68. 10.1038/ncb3272 26595383 PMC4663685

[42] Kung HN, Marks JR, Chi JT. Glutamine synthetase is a genetic determinant of cell type-specific glutamine independence in breast epithelia. PLoS Genet. 2011; 7:e1002229. 10.1371/journal.pgen.1002229 21852960 PMC3154963

[43] Berlicki Ł. Inhibitors of glutamine synthetase and their potential application in medicine. Mini Rev Med Chem. 2008; 8:869–78. 10.2174/138955708785132800 18691144

[44] Yoo HC, Yu YC, Sung Y, Han JM. Glutamine reliance in cell metabolism. Exp Mol Med. 2020; 52:1496–516. 10.1038/s12276-020-00504-8 32943735 PMC8080614

[45] Altman BJ, Stine ZE, Dang CV. From Krebs to clinic: glutamine metabolism to cancer therapy. Nat Rev Cancer. 2016; 16:619–34. 10.1038/nrc.2016.71 27492215 PMC5484415

[46] Bott AJ, Maimouni S, Zong WX. The Pleiotropic Effects of Glutamine Metabolism in Cancer. Cancers (Basel). 2019; 11:770. 10.3390/cancers11060770 31167399 PMC6627534

[47] Scalise M, Console L, Rovella F, Galluccio M, Pochini L, Indiveri C. Membrane Transporters for Amino Acids as Players of Cancer Metabolic Rewiring. Cells. 2020; 9:2028. 10.3390/cells9092028 32899180 PMC7565710

[48] Saleh T, Bloukh S, Carpenter VJ, Alwohoush E, Bakeer J, Darwish S, Azab B, Gewirtz DA. Therapy-Induced Senescence: An "Old" Friend Becomes the Enemy. Cancers (Basel). 2020; 12:822. 10.3390/cancers12040822 32235364 PMC7226427

[49] Lau L, David G. Pro- and anti-tumorigenic functions of the senescence-associated secretory phenotype. Expert Opin Ther Targets. 2019; 23:1041–51. 10.1080/14728222.2019.1565658 30616404 PMC6614023

[50] Wang B, Kohli J, Demaria M. Senescent Cells in Cancer Therapy: Friends or Foes? Trends Cancer. 2020; 6:838–57. 10.1016/j.trecan.2020.05.004 32482536

[51] Chitikova ZV, Gordeev SA, Bykova TV, Zubova SG, Pospelov VA, Pospelova TV. Sustained activation of DNA damage response in irradiated apoptosis-resistant cells induces reversible senescence associated with mTOR downregulation and expression of stem cell markers. Cell Cycle. 2014; 13:1424–39. 10.4161/cc.28402 24626185 PMC4050140

[52] Bröer A, Rahimi F, Bröer S. Deletion of Amino Acid Transporter ASCT2 (SLC1A5) Reveals an Essential Role for Transporters SNAT1 (SLC38A1) and SNAT2 (SLC38A2) to Sustain Glutaminolysis in Cancer Cells. J Biol Chem. 2016; 291:13194–205. 10.1074/jbc.M115.700534 27129276 PMC4933233

[53] Chiu M, Sabino C, Taurino G, Bianchi MG, Andreoli R, Giuliani N, Bussolati O. GPNA inhibits the sodium-independent transport system L for neutral amino acids. Amino Acids. 2017; 49:1365–72. 10.1007/s00726-017-2436-z 28516268

[54] Corti A, Dominici S, Piaggi S, Belcastro E, Chiu M, Taurino G, Pacini S, Bussolati O, Pompella A. γ-Glutamyltransferase enzyme activity of cancer cells modulates L-γ-glutamyl-p-nitroanilide (GPNA) cytotoxicity. Sci Rep. 2019; 9:891. 10.1038/s41598-018-37385-x 30696905 PMC6351548

[55] Flynn L, Barr MP, Baird AM, Smyth P, Casey OM, Blackshields G, Greene J, Pennington SR, Hams E, Fallon PG, O'Leary J, Sheils O, Finn SP. Prostate cancer-derived holoclones: a novel and effective model for evaluating cancer stemness. Sci Rep. 2020; 10:11329. 10.1038/s41598-020-68187-9 32647229 PMC7347552

[56] Locke M, Heywood M, Fawell S, Mackenzie IC. Retention of intrinsic stem cell hierarchies in carcinoma-derived cell lines. Cancer Res. 2005; 65:8944–50. 10.1158/0008-5472.CAN-05-0931 16204067

[57] Zhou Y, Yang H, Xia W, Cui L, Xu R, Lu H, Xue Z, Zhang B, Tian Z, Cao Y, Xing Z, Yin S, Wang K, et al. Isolation and identification of cancer stem cells from PC3 human prostate carcinoma cell line. Int J Clin Exp Pathol. 2017; 10:8377–82. 31966689 PMC6965485

[58] Weyandt JD, Thompson CB, Giaccia AJ, Rathmell WK. Metabolic Alterations in Cancer and Their Potential as Therapeutic Targets. Am Soc Clin Oncol Educ Book. 2017; 37:825–32. 10.14694/EDBK_175561 28561705 PMC5954416

[59] Lin X, Xiao Z, Chen T, Liang SH, Guo H. Glucose Metabolism on Tumor Plasticity, Diagnosis, and Treatment. Front Oncol. 2020; 10:317. 10.3389/fonc.2020.00317 32211335 PMC7069415

[60] Varghese E, Samuel SM, Líšková A, Samec M, Kubatka P, Büsselberg D. Targeting Glucose Metabolism to Overcome Resistance to Anticancer Chemotherapy in Breast Cancer. Cancers (Basel). 2020; 12:2252. 10.3390/cancers12082252 32806533 PMC7464784

[61] Laussel C, Léon S. Cellular toxicity of the metabolic inhibitor 2-deoxyglucose and associated resistance mechanisms. Biochem Pharmacol. 2020; 182:114213. 10.1016/j.bcp.2020.114213 32890467

[62] Birsoy K, Possemato R, Lorbeer FK, Bayraktar EC, Thiru P, Yucel B, Wang T, Chen WW, Clish CB, Sabatini DM. Metabolic determinants of cancer cell sensitivity to glucose limitation and biguanides. Nature. 2014; 508:108–12. 10.1038/nature13110 24670634 PMC4012432

[63] Shiratori R, Furuichi K, Yamaguchi M, Miyazaki N, Aoki H, Chibana H, Ito K, Aoki S. Glycolytic suppression dramatically changes the intracellular metabolic profile of multiple cancer cell lines in a mitochondrial metabolism-dependent manner. Sci Rep. 2019; 9:18699. 10.1038/s41598-019-55296-3 31822748 PMC6904735

[64] Maher JC, Wangpaichitr M, Savaraj N, Kurtoglu M, Lampidis TJ. Hypoxia-inducible factor-1 confers resistance to the glycolytic inhibitor 2-deoxy-D-glucose. Mol Cancer Ther. 2007; 6:732–41. 10.1158/1535-7163.MCT-06-0407 17308069

[65] Zhong D, Xiong L, Liu T, Liu X, Liu X, Chen J, Sun SY, Khuri FR, Zong Y, Zhou Q, Zhou W. The glycolytic inhibitor 2-deoxyglucose activates multiple prosurvival pathways through IGF1R. J Biol Chem. 2009; 284:23225–33. 10.1074/jbc.M109.005280 19574224 PMC2749096

[66] Nguyen TL, Durán RV. Glutamine metabolism in cancer therapy. Cancer Drug Resist. 2018; 1:126–38. 10.20517/cdr.2018.08

[67] Furusawa A, Miyamoto M, Takano M, Tsuda H, Song YS, Aoki D, Miyasaka N, Inazawa J, Inoue J. Ovarian cancer therapeutic potential of glutamine depletion based on GS expression. Carcinogenesis. 2018; 39:758–66. 10.1093/carcin/bgy033 29617730

[68] Sappington DR, Siegel ER, Hiatt G, Desai A, Penney RB, Jamshidi-Parsian A, Griffin RJ, Boysen G. Glutamine drives glutathione synthesis and contributes to radiation sensitivity of A549 and H460 lung cancer cell lines. Biochim Biophys Acta. 2016; 1860:836–43. 10.1016/j.bbagen.2016.01.021 26825773 PMC4768472

[69] Pavlova NN, Hui S, Ghergurovich JM, Fan J, Intlekofer AM, White RM, Rabinowitz JD, Thompson CB, Zhang J. As Extracellular Glutamine Levels Decline, Asparagine Becomes an Essential Amino Acid. Cell Metab. 2018; 27:428–38.e5. 10.1016/j.cmet.2017.12.006 29337136 PMC5803449

[70] Bott AJ, Shen J, Tonelli C, Zhan L, Sivaram N, Jiang YP, Yu X, Bhatt V, Chiles E, Zhong H, Maimouni S, Dai W, Velasquez S, et al. Glutamine Anabolism Plays a Critical Role in Pancreatic Cancer by Coupling Carbon and Nitrogen Metabolism. Cell Rep. 2019; 29:1287–98.e6. 10.1016/j.celrep.2019.09.056 31665640 PMC6886125

[71] Zhu Y, Li T, Ramos da Silva S, Lee JJ, Lu C, Eoh H, Jung JU, Gao SJ. A Critical Role of Glutamine and Asparagine γ-Nitrogen in Nucleotide Biosynthesis in Cancer Cells Hijacked by an Oncogenic Virus. mBio. 2017; 8:e01179–17. 10.1128/mBio.01179-17 28811348 PMC5559638

[72] DeBerardinis RJ, Cheng T. Q's next: the diverse functions of glutamine in metabolism, cell biology and cancer. Oncogene. 2010; 29:313–24. 10.1038/onc.2009.358 19881548 PMC2809806

[73] Dimri GP, Lee X, Basile G, Acosta M, Scott G, Roskelley C, Medrano EE, Linskens M, Rubelj I, Pereira-Smith O. A biomarker that identifies senescent human cells in culture and in aging skin in vivo. Proc Natl Acad Sci U S A. 1995; 92:9363–67. 10.1073/pnas.92.20.9363 7568133 PMC40985

