# Supplementary material for: Glutamine promotes escape from therapy-induced senescence in tumor cells
Source: Aging (Albany NY). 2021 Sep 7;13(17):20962–91. doi: 10.18632/aging.203495 (PMC8457561; doi:10.18632/aging.203495)
Supplement: Supplementary Figures [file aging-13-203495-s001.pdf]

SUPPLEMENTARY FIGURES

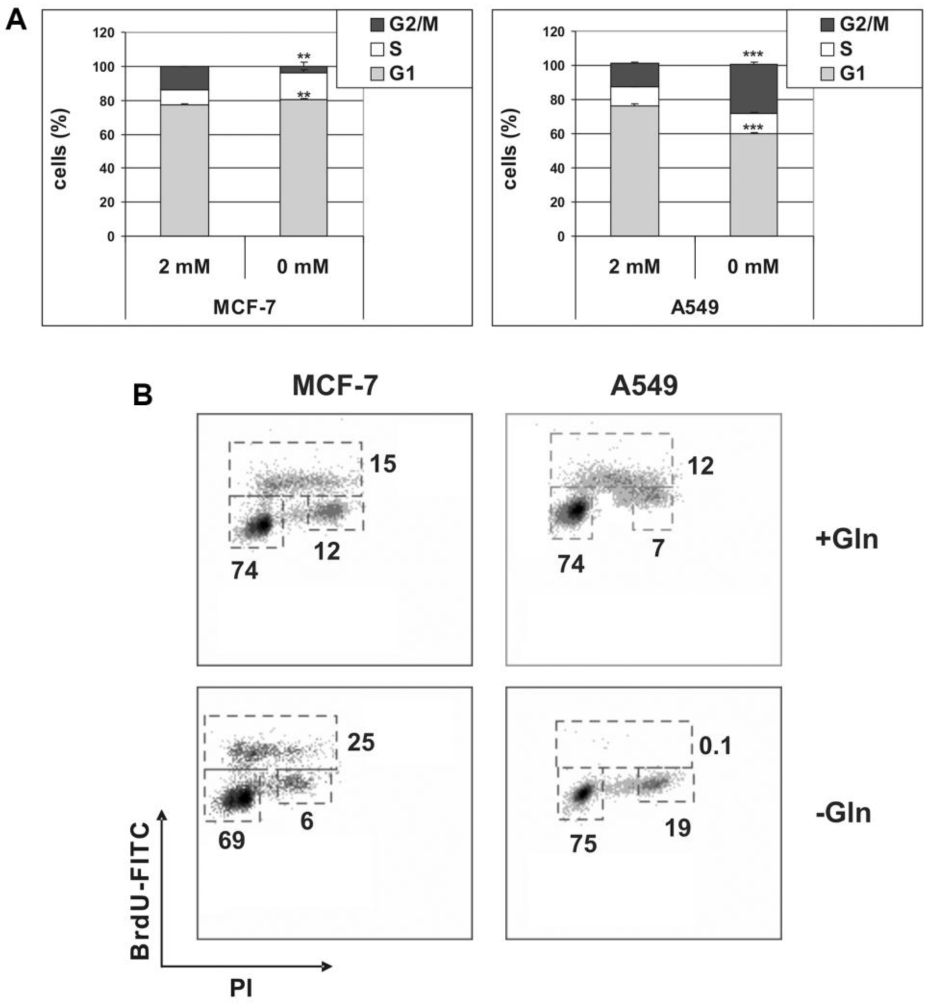

C

sub-G1 (%)	+Gln	- Gln
MCF-7	0.63 ± 0.23	0.23 ± 0.07
A549	1.23 ± 0.06	0.93 ± 0.06

Supplementary Figure 1. Cell cycle distribution in senescent carcinoma cells. (**A**) Cell cycle distribution in MCF-7 and A549 cells grown in Gln-supplemented or Gln-deprived conditions for 72 h, analyzed by flow cytometry following propidium iodide staining. The percentage of cells in G1, S, or G2/M phase is shown. (**B**) Representative flow cytometric data. Proliferating MCF-7 and A549, grown in Gln-supplemented (+Gln) or Gln-deprived (−Gln) conditions for 72 h, were incubated with BrdU, for 30 min. The number of BrdU-labelled cells was determined by flow cytometry following propidium iodide staining. The percentage of BrdU-labelled cells and cells in G1 and G2/M phase is shown. (**C**) Proliferating MCF-7 and A549, grown in Gln-supplemented (+Gln) or Gln-deprived (−Gln) conditions for 72 h, were stained with propidium iodide and analyzed by flow cytometry. The percentage of cells with sub-G1 DNA content is reported.

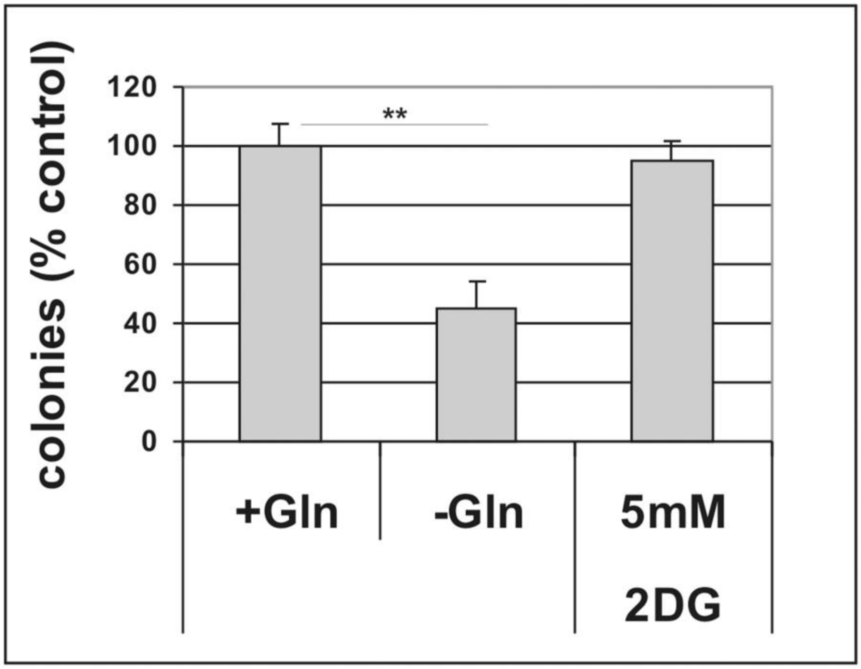

Supplementary Figure 2. The effect of glutamine deprivation on TIS escape. Cisplatin-induced senescent MCF-7 cells were grown in the presence (+Gln) or in the absence (−Gln) of 2 mM glutamine or in the presence of 2-deoxyglucose (2DG). Colonies that evaded the senescent growth arrest were stained and counted. Data are mean ± S.D. of two independent experiments.

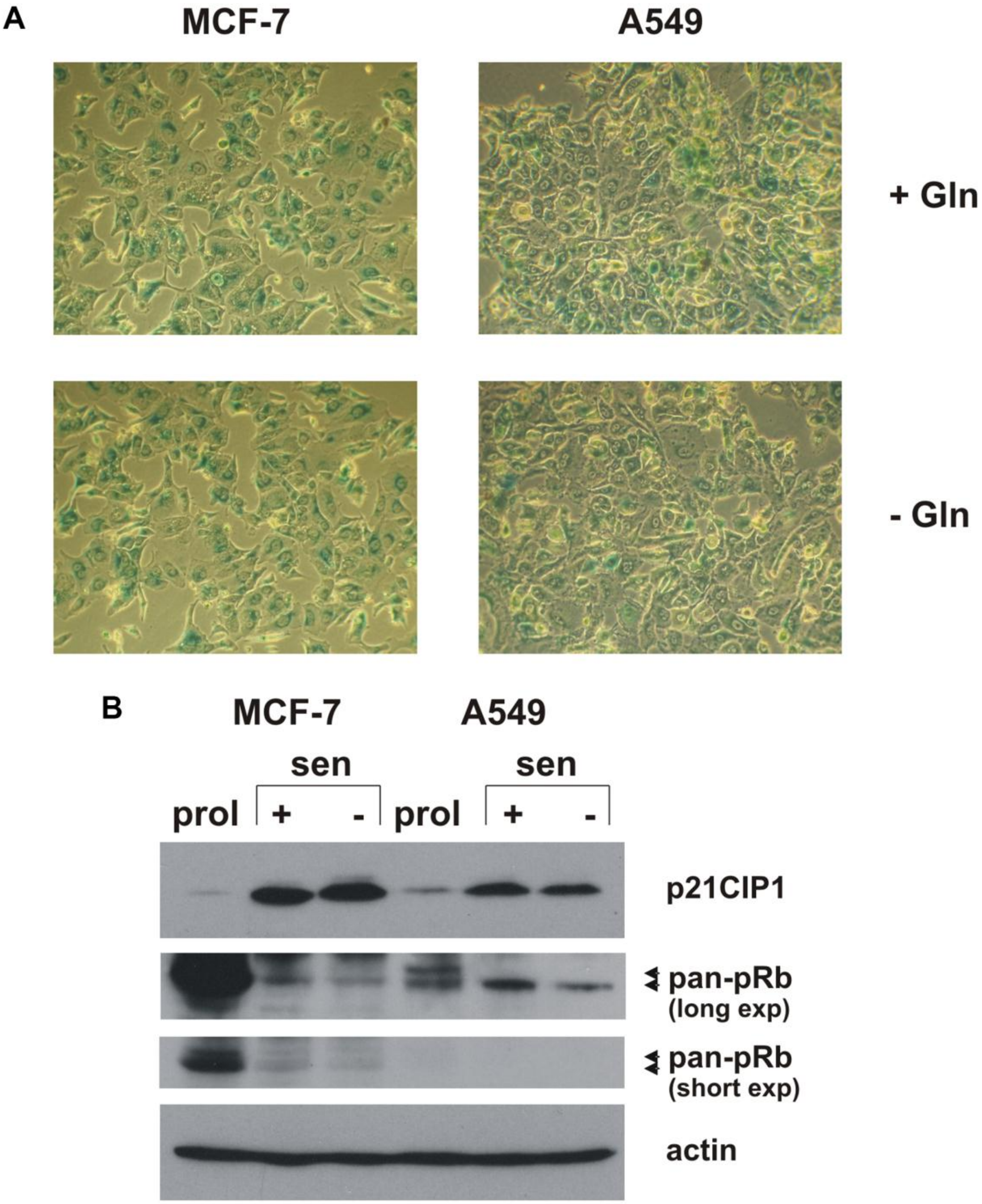

Supplementary Figure 3. Glutamine withdrawal did not alter development of TIS. MCF-7 and A549 cells were treated with doxorubicin for 72 h. Cells were extensively washed, released in complete medium to allow for cell attachment, and thereafter maintained in either complete medium (+Gln) or in Gln-deprived medium (−Gln), and analyzed 7 days after release from the drug. (**A**) Morphological alterations and SA-β-gal staining. Phase contrast microscopy images were captured using Canon powershot G6 camera at 10× magnification, 6× digital zoom. (**B**) Accumulation of $p21^{CIP1}$ and hypophosphorylated pRb protein in drug-induced senescent cells maintained in either complete medium (+) or in Gln-deprived medium (−).

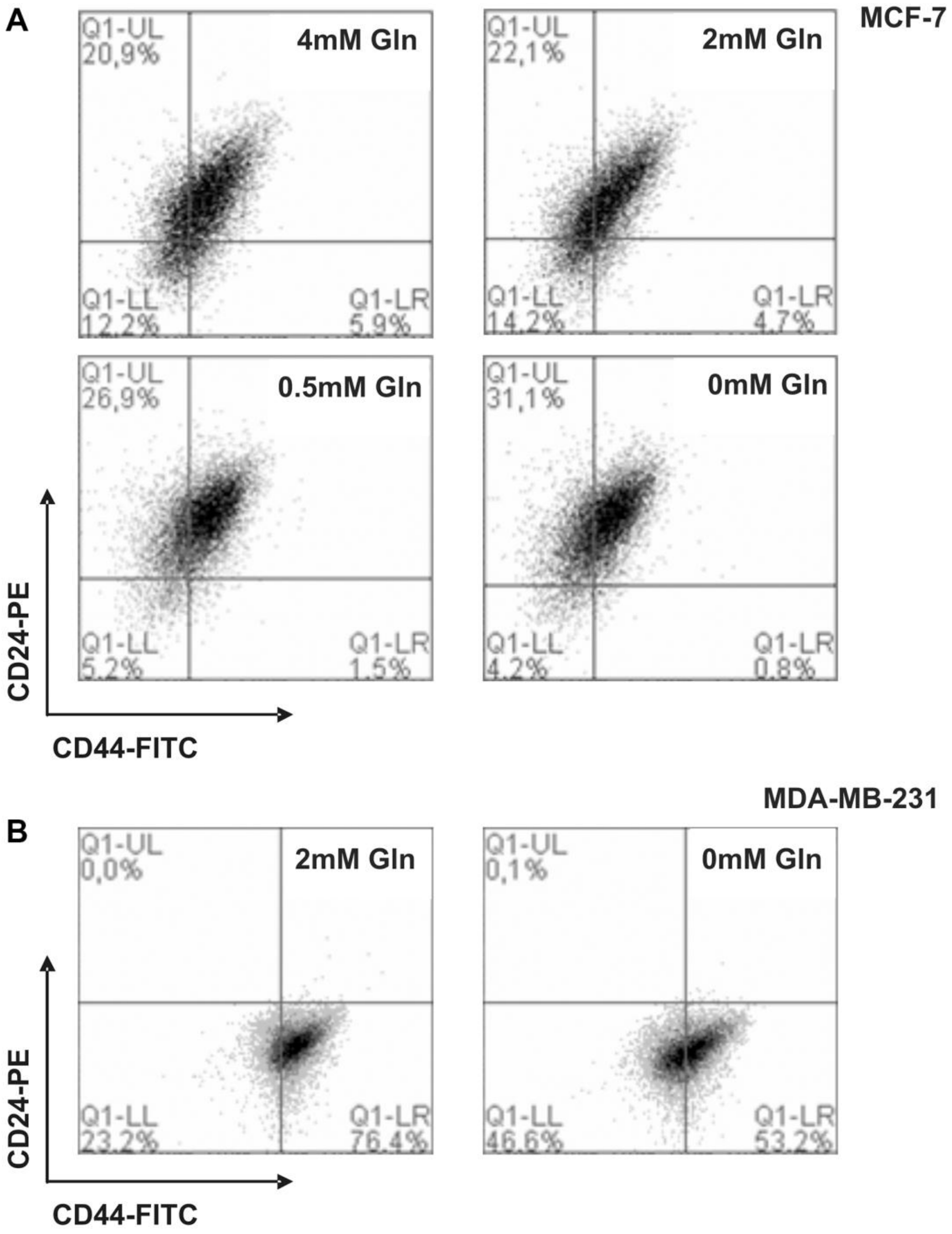

Supplementary Figure 4. Representative flow cytometric data. (**A**) MCF-7 cells were grown for 48 hours in media with different glutamine concentrations. Expression of CD44 and CD24 was analyzed by flow cytometry. (**B**) MDA-MB-231 cells were grown for 48 hours in media with different glutamine concentrations. Expression of CD44 and CD24 was analyzed by flow cytometry.

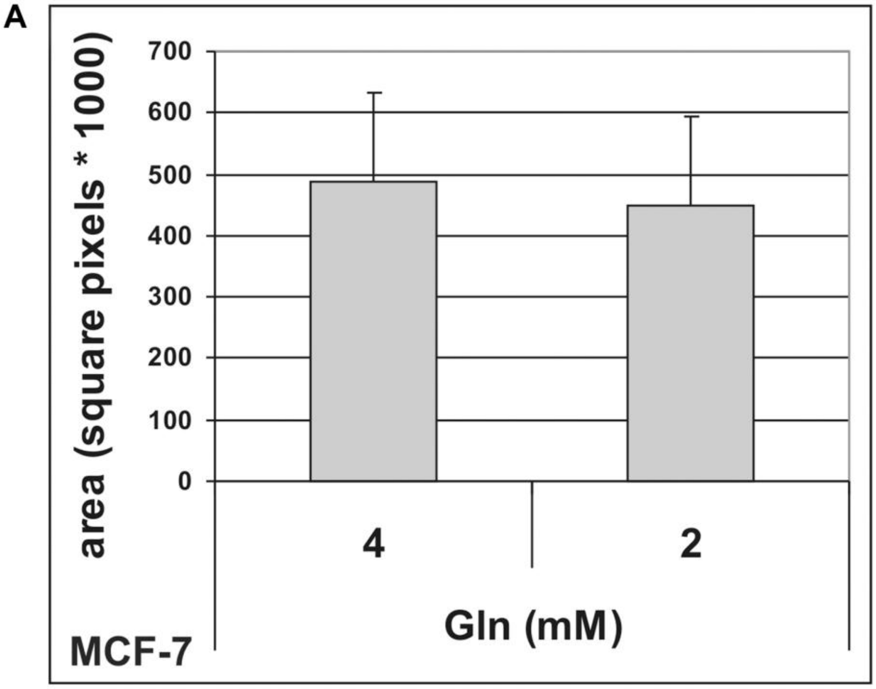

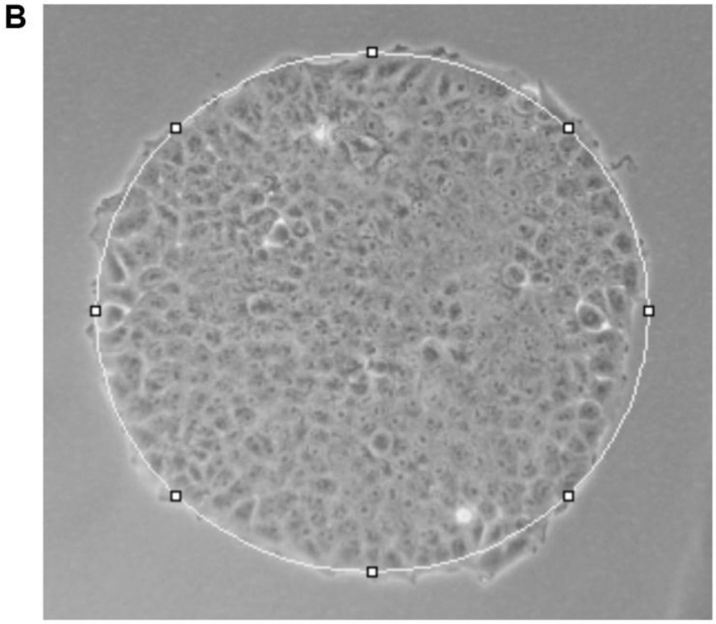

Supplementary Figure 5. MCF-7 cells were cultured at low density in the presence of 4 mM or 2 mM glutamine. (**A**) After 7 days of culture, the size of holoclone was estimated in more than 50 colonies. (**B**) Representative phase contrast microscopy image of holoclone analyzed with area tool of ImageJ software.

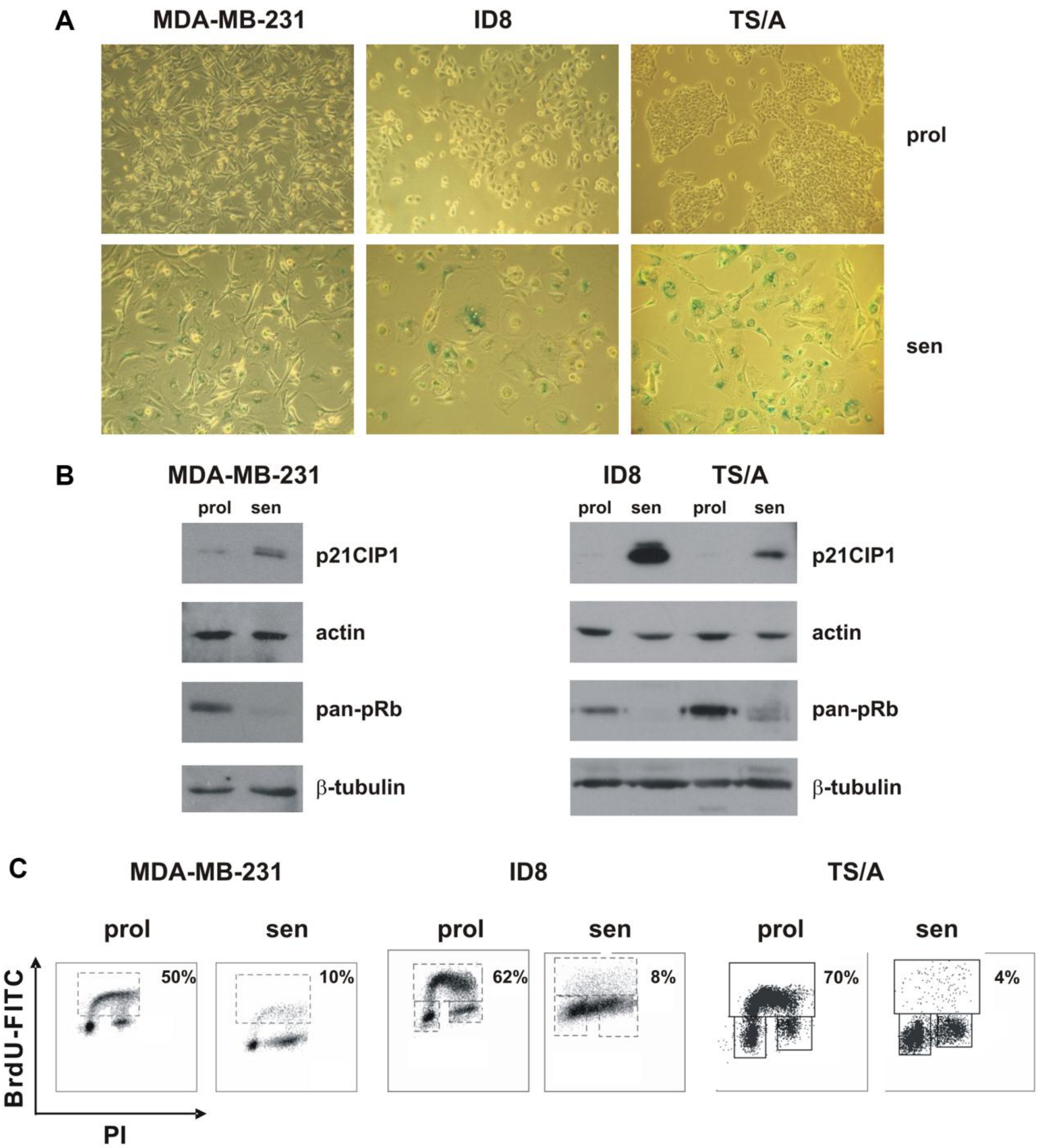

Supplementary Figure 6. Premature senescence in MDA-MB-231, ID8 and TS/A cells. Cells were treated with doxorubicin for 72 h. Cells were extensively washed and analyzed 5 days after release from the drug. (**A**) Proliferating cells and doxorubicin-induced senescent cells were stained to detect SA-β-gal activity. Phase contrast microscopy images were captured using Canon powershot G6 camera at 10× magnification, 6× digital zoom. (**B**) Accumulation of $p21^{CIP1}$ and hypophosphorylated pRb protein in drug-induced senescent cells. (**C**) Representative flow cytometric data. Proliferating and senescent cells were incubated with BrdU for 30 min and 1 hour, respectively. The number of BrdU-labelled cells was determined and the percentage is shown in the chart.

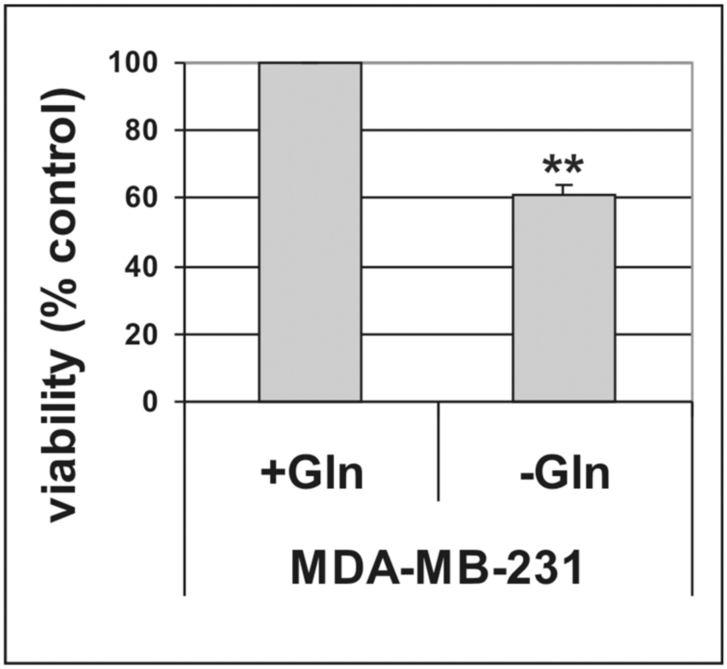

Supplementary Figure 7. Effect of glutamine deprivation on cells viability. MDA-MB-231 cells were grown for 72 hours in the presence (+Gln) or in the absence (−Gln) of glutamine. Cell viability was determined by MTS assay. Data are mean ± S.D. of two independent experiments.

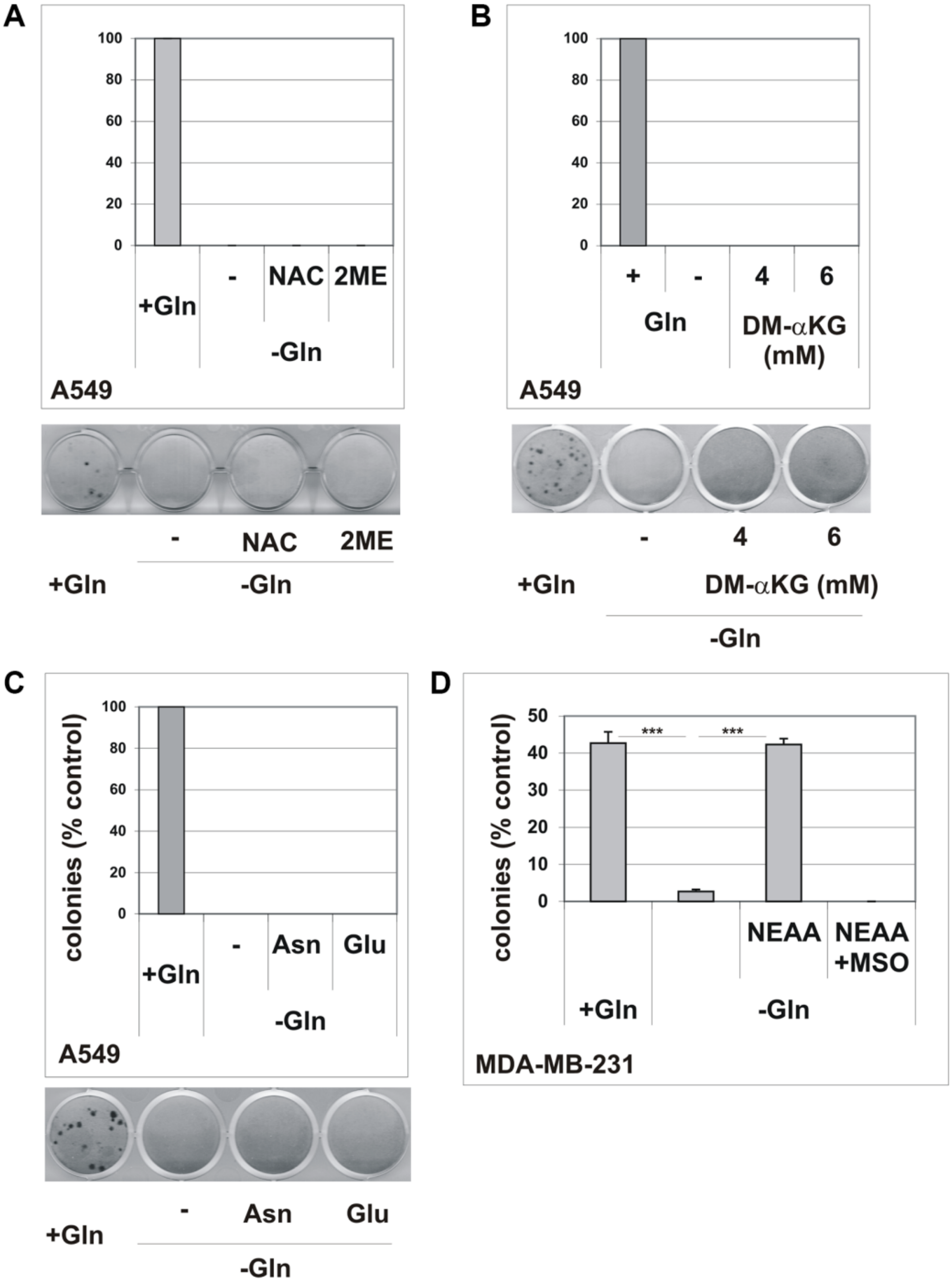

Supplementary Figure 8. Rescue experiments with glutamine-derived metabolites. (**A**) Doxorubicin-induced senescent A549 cells were grown with (+Gln) or without (−Gln) glutamine plus either 1 mM NAC or 25 µM 2ME. Colonies that evaded the senescent growth arrest were stained and counted. The data shown here represent three experiments exhibiting similar effects. A representative image of the colony escape assay is shown. (**B**) Doxorubicin-induced senescent A549 cells were grown with (+Gln), or without (−Gln) glutamine plus DM-αKG (4 or 6 mM). Colonies that evaded the senescent growth arrest were stained and counted. The data shown here represent three experiments exhibiting similar effects. A representative image of the colony escape assay is shown. (**C**) Doxorubicin-induced senescent A549 cells were grown with (+Gln) or without (−Gln) glutamine plus either 2 mM asparagine (Asn) or 2 mM glutamate (Glu). Colonies that evaded the senescent growth arrest were stained and counted. The data shown here represent three experiments exhibiting similar effects. A representative image of the colony escape assay is shown. (**D**) Doxorubicin-induced senescent MDA-MB-231 cells were grown with (+Gln) or without (−Gln) glutamine plus NEAA, in the presence or in the absence of 2 mM MSO. Colonies that evaded the senescent growth arrest were stained and counted. The data shown here represent three experiments exhibiting similar effects.

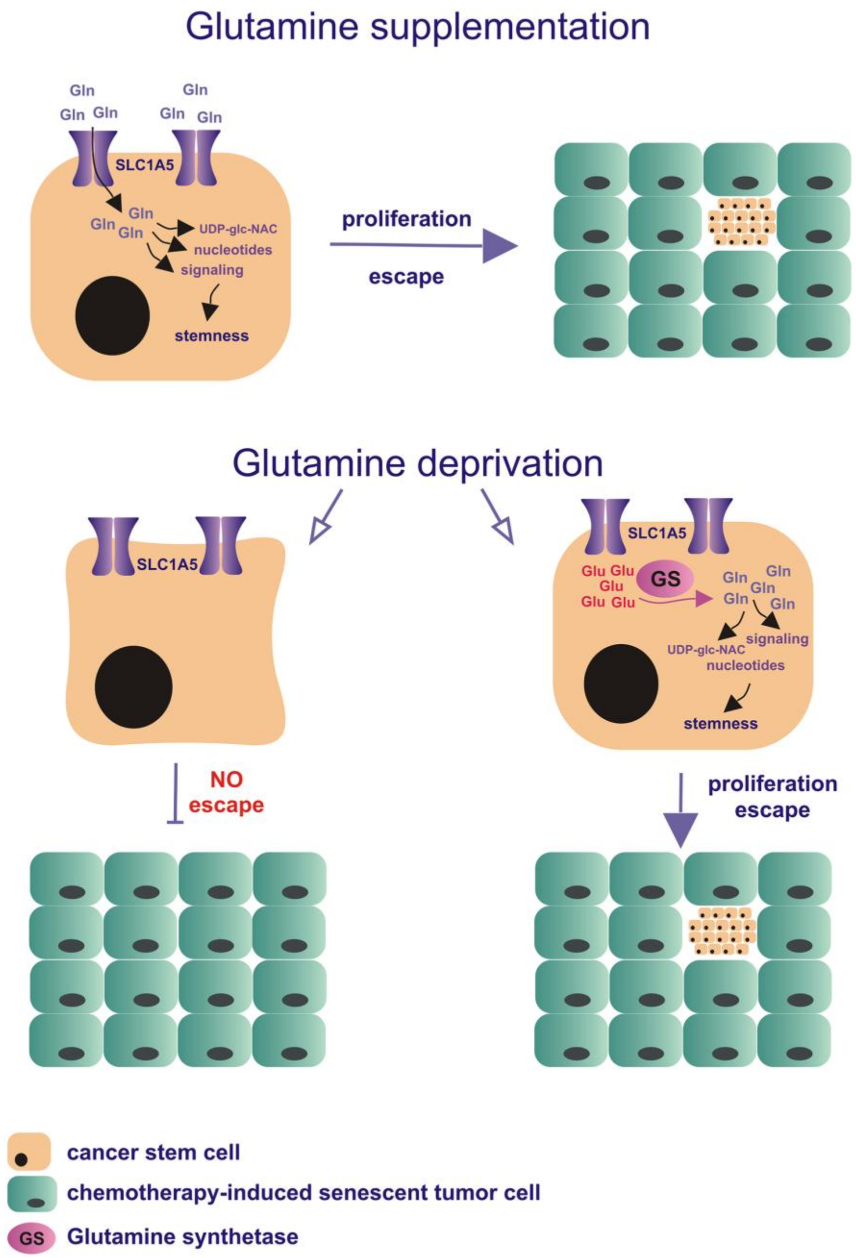

Supplementary Figure 9. Proposed model of glutamine metabolism and the role of GS in TIS escape. Glutamine metabolism supports cancer cell stemness allowing escape from therapy-induced senescence. Under glutamine-limited conditions cancer cells are unable to escape from TIS, but GS upregulation rescues TIS escape in the absence of exogenous glutamine.
